# Genome sequencing of the multicellular alga *Astrephomene* provides insights into convergent evolution of germ-soma differentiation

**DOI:** 10.1038/s41598-021-01521-x

**Published:** 2021-11-22

**Authors:** Shota Yamashita, Kayoko Yamamoto, Ryo Matsuzaki, Shigekatsu Suzuki, Haruyo Yamaguchi, Shunsuke Hirooka, Yohei Minakuchi, Shin-ya Miyagishima, Masanobu Kawachi, Atsushi Toyoda, Hisayoshi Nozaki

**Affiliations:** 1 Department of Biological Sciences, Graduate School of Science, The University of Tokyo, 7-3-1 Hongo, Bunkyo, Tokyo, 113-0033 Japan; 2 Biodiversity Division, National Institute for Environmental Studies, 16-2 Onogawa, Tsukuba, Ibaraki 305-8506 Japan; 3 Faculty of Life and Environmental Sciences, University of Tsukuba, 1-1-1 Tennodai, Tsukuba, Ibaraki 305-8572 Japan; 4 Department of Gene Function and Phenomics, National Institute of Genetics, 1111 Yata, Mishima, Shizuoka 411-8540 Japan; 5 Center for Information Biology, National Institute of Genetics, 1111 Yata, , Mishima, Shizuoka 411-8540 Japan; 6 Present Address: Department of Gene Function and Phenomics, National Institute of Genetics, 1111 Yata, Mishima, Shizuoka 411-8540 Japan; 7 Present Address: Department of Chemical and Biological Sciences, Faculty of Science, Japan Women’s University, Bunkyo-ku, Tokyo, 112-8681 Japan

**Keywords:** Evolution, Molecular biology

## Abstract

Germ-soma differentiation evolved independently in many eukaryotic lineages and contributed to complex multicellular organizations. However, the molecular genetic bases of such convergent evolution remain unresolved. Two multicellular volvocine green algae, *Volvox* and *Astrephomene*, exhibit convergent evolution of germ-soma differentiation. The complete genome sequence is now available for *Volvox*, while genome information is scarce for *Astrephomene*. Here, we generated the de novo whole genome sequence of *Astrephomene gubernaculifera* and conducted RNA-seq analysis of isolated somatic and reproductive cells. In *Volvox*, tandem duplication and neofunctionalization of the ancestral transcription factor gene (*RLS1*/*rlsD*) might have led to the evolution of *regA*, the master regulator for *Volvox* germ-soma differentiation. However, our genome data demonstrated that *Astrephomene* has not undergone tandem duplication of the *RLS1*/*rlsD* homolog or acquisition of a *regA*-like gene. Our RNA-seq analysis revealed the downregulation of photosynthetic and anabolic gene expression in *Astrephomene* somatic cells, as in *Volvox*. Among genes with high expression in somatic cells of *Astrephomene*, we identified three genes encoding putative transcription factors, which may regulate somatic cell differentiation. Thus, the convergent evolution of germ-soma differentiation in the volvocine algae may have occurred by the acquisition of different regulatory circuits that generate a similar division of labor.

## Introduction

The evolution from unicellular to multicellular organisms is one of the major transitions in evolution, during which individual lower-level units are integrated into a higher-level unit^1,2^. Multicellular organisms independently evolved from unicellular ancestors at least 25 times in a broad range of prokaryotic and eukaryotic branches^3^. Moreover, many multicellular lineages have acquired complex features for a multicellular lifestyle, such as connections between individual cells by cell adhesion molecules and/or an extracellular matrix (ECM) for formation of a multicellular body, functional differentiation between cell groups for increase of fitness as individuals, and three-dimensional body plans or structures^4^. Most multicellular lineages have lost the extant intermediates between complex multicellular organisms and their closest unicellular ancestors, which hampers studies of the initial steps in the evolution of multicellular complexity.

**Figure 1:**
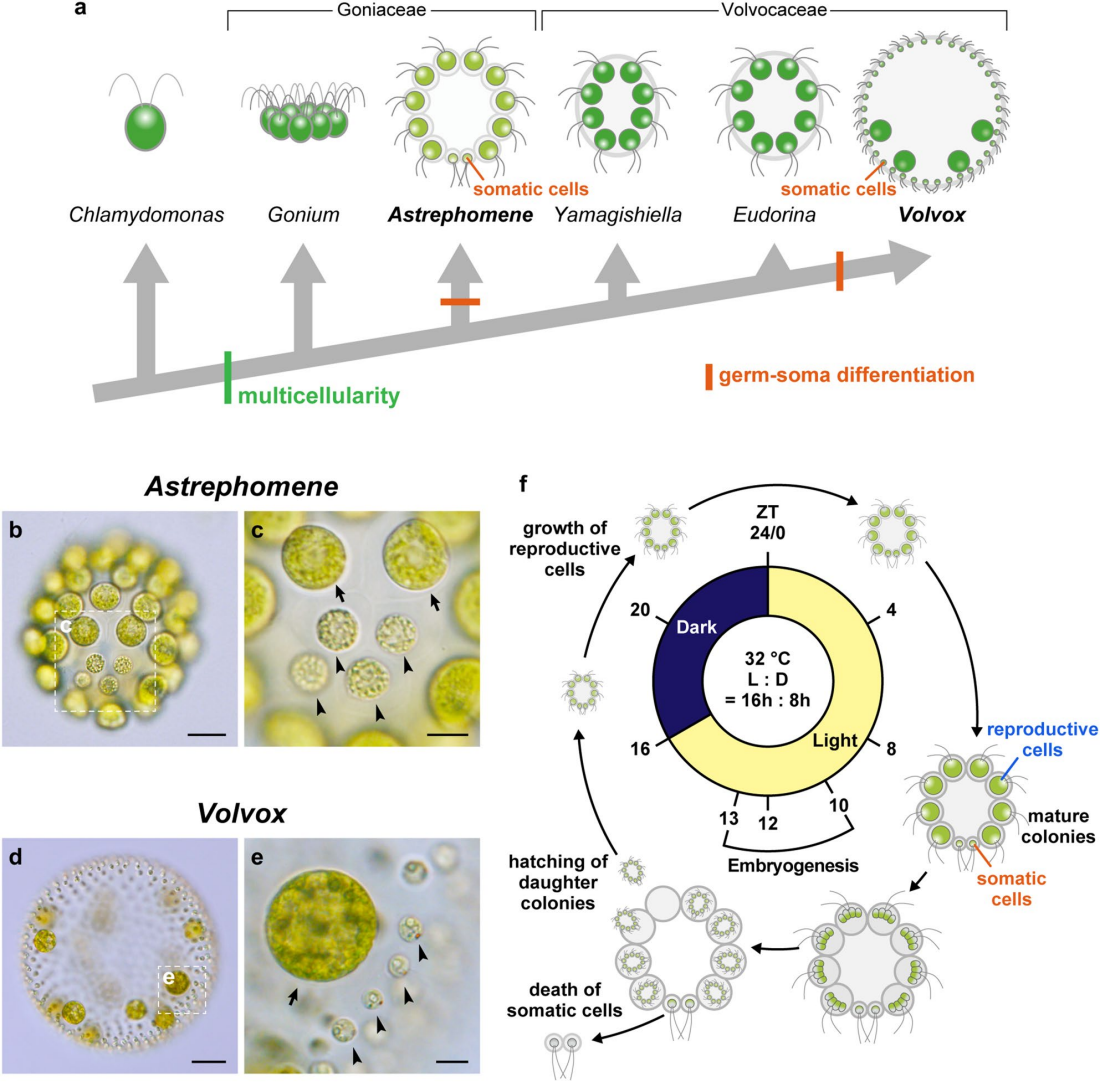
Convergent evolution of germ-soma differentiation in *Astrephomene* and *Volvox*. (**a**) Schematic representation of the phylogenetic relationships of the volvocine algae and the parallel evolution of germ-soma differentiation. Volvocine algae include unicellular *Chlamydomonas*, multicellular *Volvox* and various intermediate forms and a suitable model for research into the evolution of multicellularity^5^. Germ-soma differentiation are thought to have evolved twice or more independently within this group^24,25^. *Astrephomene* have acquired somatic cells independently of Volvocaceae. The phylogenetic relationships are based on the present phylogenomic analysis (Supplementary Fig. 4) and a previous report^38^. The genomes of all genera except for *Astrephomene* have been sequenced in previous studies^7–11^. (**b**) A vegetative colony of *Astrephomene gubernaculifera.* Scale bar = 20 μm. (**c**) Four posterior somatic cells (arrowheads) in *A*. *gubernaculifera*. Compared to reproductive cells (arrows), somatic cells are small and pale colored. Scale bar = 10 μm. (**d**) A vegetative colony of *Volvox carteri*. Scale bar = 50 μm. (**e**) Somatic cells (arrowheads) and gonidia (reproductive cells, arrow) in *V*. *carteri*. While somatic cells are biflagellate, gonidia have no flagella. Scale bar = 10 μm. (**f**) Schematic diagram of the asexual life cycle of *A*. *gubernaculifera* in the synchronous culture. The asexual life cycle is completed in approximately 24 h, and the culture is highly synchronized with the light–dark cycle. While reproductive cells undergo cell growth and embryogenesis to form daughter colonies, somatic cells do not undergo obvious growth or embryogenesis and reach cell death. *ZT* Zeitgeber time.

One of the best model groups for examining the initial evolution of multicellularity is volvocine green algae^5^ (Fig. 1a). This group, which consists of *Volvox* and its closely related genera, includes organisms of various intermediate stages of multicellular complexity, ranging from unicellular *Chlamydomonas* to multicellular *Volvox* with germ-soma division of labor in the expanded multicellular body (spheroid). The multicellular groups are estimated to have diverged from unicellular ancestors approximately 200 Mya^6^, which is more recent than that of metazoans or land plants. Genome studies of this group have been intensively promoted in recent years, and whole nuclear sequence data of some representative genera are currently available^7–11^. Diverse molecular biology tools are also available in *Chlamydomonas reinhardtii* and *Volvox carteri*^12,13^ and are potentially applicable to other volvocine species^14–16^. Thus, this group is a suitable model for the stepwise evolution of multicellular complexity at both the genetic and genomic level.

Among the multicellular traits observed in the volvocine green algae, differentiation between somatic cells and reproductive (germline) cells has been a central topic of evolutionary biology for more than a century, since Weismann pointed out the germ-soma differentiation of *Volvox* in his Germ-plasm theory^17^. In general, the evolution of somatic cells, which specialize for altruistic, non-reproductive functions such as motility or molecular transport^18–20^, enhances the reproduction of other (reproductive) cells and increases fitness at the organismal level^21^. On the other hand, the evolution of specialized reproductive cells must have extended the reproductive timescale to allow natural selection to operate over the longer timescale of a multicellular life cycle, enabling the evolution of developmental programs for multicellular complexity^22^. Specialization between somatic and reproductive cells tends to occur as the size of the individual and/or the number of cells per individual increase^23^. Consistent with theoretical studies, germ-soma differentiation has evolved repeatedly in volvocine genera with large numbers of cells^6,24^. Among these, two phylogenetically separate lineages, *Volvox* and *Astrephomene*, have evolved germ-soma differentiation^5,24,25^ (Fig. 1).

*Volvo*x exhibits the most apparent germ-soma division of labor within the volvocine lineage^5^ (Fig. 1d,e). A vegetative or asexual spheroid of *V*. *carteri* consists of ~ 2000 somatic cells and ~ 16 reproductive cells (gonidia). The somatic cells are small, biflagellate, and distributed throughout the surface of the spheroid, whereas the gonidia are much larger than somatic cells and have no flagella (Fig. 1e). Molecular genetic studies of germ-soma differentiation in *V*. *carteri* have identified the master regulatory gene of somatic cells, *regA*^26^, and its evolutionary origin^9,27–29^. Previous studies of *V*. *carteri* have also revealed specialization of somatic cells for flagellar motility and ECM biosynthesis and the specialization of reproductive cells for photosynthesis and anabolism, at the transcriptomic level^30–32^.

*Astrephomene* is another volvocine genus with germ-soma differentiation (Fig. 1b,c). Two or four somatic cells occur at the posterior pole of a 32- or 64-celled vegetative colony in *Astrephomene*^33,34^. Unlike *Volvox*, both reproductive and somatic cells in *Astrephomene* colonies have flagella and they are almost identical in cell size in newly formed colonies^35^. As the colonies grow, however, the reproductive cells become larger than the somatic cells in size (Fig. 1f)^35^. In contrast to *Volvox*, the molecular genetic bases involved in the germ-soma differentiation of *Astrephomene* have not yet been investigated, and no genome data have been established. Therefore, it was unknown whether *Astrephomene* has also acquired *regA* or other regulators or how cell growth is regulated between cell types. Moreover, though *Astrephomene* is photoheterotrophic and requires acetate or some carboxylic acid as a carbon source for growth^36^, whether the heterotrophy of *Astrephomene* affected the evolution of germ-soma differentiation is also unknown.

The objective of this study was to examine the molecular genetic bases of the convergent evolution of germ-soma differentiation, by focusing on *Volvox* and *Astrephomene* within the volvocine algae. Here, we generated the de novo whole nuclear genome of *Astrephomene gubernaculifera* and performed RNA-seq analysis of isolated somatic and reproductive cells of this species. Our results demonstrated that germ-soma differentiation with similar gene expression patterns may have evolved in *Volvox* and *Astrephomene* via the acquisition of different molecular genetic bases.

## Results and discussion

### De novo genome sequence of *Astrephomene gubernaculifera*

The de novo whole nuclear genome of *A*. *gubernaculifera* strain NIES-4017 was constructed by assembling a combination of long and short sequencing reads into contigs (see “Methods”). The genome assembly with 103,829,650 bp from 206 gap-free contigs and N50 of 1,506,991 bp was obtained. The quality of assembly was verified using BUSCO^37^, which showed the presence of 97.1% genes in the reference dataset (see “Methods”). Moreover, gene prediction based on RNA-seq data identified 13,713 protein-coding gene models of genome assembly in *A*. *gubernaculifera*. The genome size and number of genes in *A*. *gubernaculifera* were slightly smaller than those of other volvocine algae (Supplementary Table 1). GC content of *A*. *gubernaculifera* was 59.9%, which is intermediate between *C*. *reinhardtii* (64.1%) and *V*. *carteri* (56.1%). Overall, the characteristics of the nuclear genome in *A*. *gubernaculifera* did not differ greatly from those in other volvocine algae. The plastid genome (414,859 bp) and mitochondrial genome (33,967 bp) were also sequenced and annotated (Supplementary Fig. 1).

The analysis of orthology of the genes in four volvocine genomes (*C. reinhardtii*, *Gonium pectorale*, *V. carteri* and *A. gubernaculifera*) showed that more than a half of orthogroups (putative sets of orthologs or gene families) were shared within the four volvocine species (Supplementary Fig. 2). On the other hand, *A*. *gubernaculifera* genome lacked many orthogroups shared in three other volvocine species (Supplementary Fig. 2a), which might have resulted in the relatively small number of genes in *A*. *gubernaculifera* genome (Supplementary Table 1). The synonymous substitution ratios (dS) of conserved orthologs are totally higher in *V*. *carteri* than those in *A*. *gubernaculifera* and *G*. *pectorale*, in contrast to the similarity in non-synonymous substitution ratio (dN) (Supplementary Fig. 3, Supplementary Methods). Phylogenomic analysis using 371 orthologs from the current nuclear genome data of *A*. *gubernaculifera* and genome data of other volvocine algae (see Supplementary Methods) positioned *Astrephomene* as a sister group of Volvocaceae (Supplementary Fig. 4), which is consistent with the recent phylotranscriptomic analysis of volvocine species^38^.

### *RLS1*/*rlsD* homolog and other VARL genes

The differentiation of somatic cells in *V*. *carteri* is controlled by *regA*, which encodes a putative transcription factor with a DNA-binding SAND-like domain (called the VARL domain), and is expressed only in somatic cells^26^. *regA* is arranged tandemly with closely related paralogs with VARL domains (*rlsA*, *rlsB*, and *rlsC*) in the *V. carteri* genome, which is called the *reg* cluster^27^. The *reg* cluster is derived from tandem duplication of another VARL domain-containing gene (*RLS1*/*rlsD*) at the common ancestor of Volvocaceae, and one of the duplicated genes, *regA*, has become the master regulator of somatic cells in *V*. *carteri*^9,28,29^. *C*. *reinhardtii* also has *RLS1*^27^, whose function may be response to environmental stresses^39,40^.

**Figure 2:**
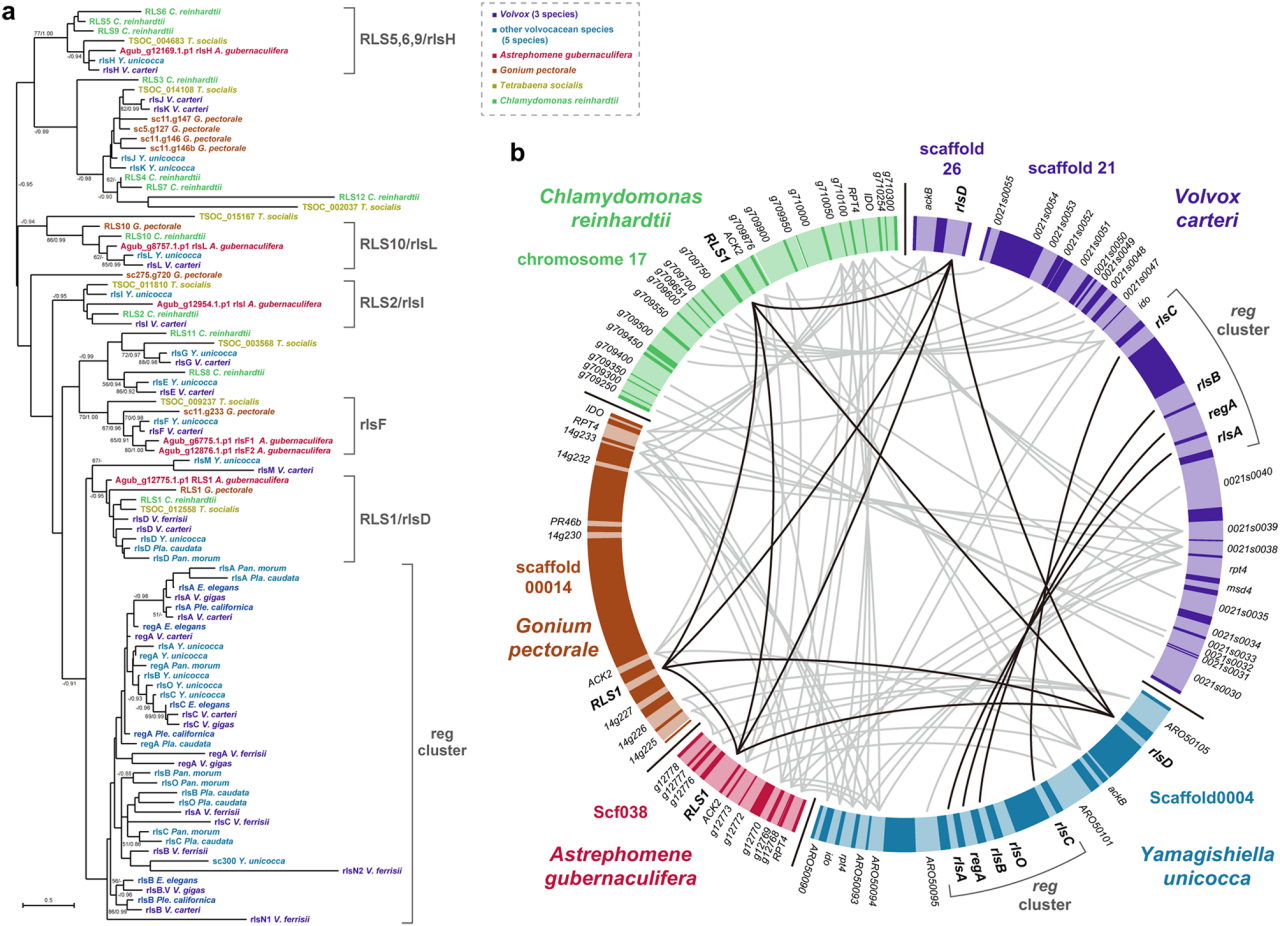
Analysis of *RLS1*/*rlsD* orthologs and VARL genes in volvocine green algae. (**a**) Maximum-likelihood phylogenetic tree of VARL domain containing protein among volvocine algae. The 87 amino acid positions of 6 VARL genes in *Astrephomene gubernaculifera* as well as all known VARL genes identified in previous studies^10,29^ were subjected to maximum-likelihood analysis and Bayesian interference (LG + I + G model). The bootstrap values from maximum-likelihood (≥ 50%, left) and posterior probabilities in Bayesian interference (≥ 0.90, right) are shown at branches. The colors indicate species according to the color key at the upper right. (**b**) Synteny of *Chlamydomonas reinhardtii*, *Gonium pectorale*, *A*. *gubernaculifera*, *Yamagishiella unicocca* and *Volvox carteri*. The synteny of *RLS1*/*rlsD* orthologs and *reg* cluster genes is indicated with black lines, and the synteny of other genes are indicated with grey lines based on BLASTP search (Supplementary Methods).

Homologs of *regA* and other genes with the VARL domain were searched in the genome of *A*. *gubernaculifera*. Six VARL genes were found in *A*. *gubernaculifera*. Phylogenetic analysis of VARL genes within volvocine algae assigned them to the *RLS1*/*rlsD* clade; *RLS5*, *6*, *9*/*rlsH* clade; *RLS10*/*rlsL* clade; *RLS2*/*rlsI* clade; and *rlsF* clade (Fig. 2a). Moreover, gene synteny including the *A*. *gubernaculifera RLS1*/*rlsD* gene was similar to that in other volvocine algae (Fig. 2b). No *A*. *gubernaculifera* homolog was included in the *reg* cluster clade (Fig. 2a), and no tandem duplication of an *RLS1*/*rlsD* ortholog was found in the *A*. *gubernaculifera* genome (Fig. 2b), unlike species in Volvocaceae^28,29^. These results suggest that *Astrephomene* may have evolved another molecular basis to differentiate somatic cells.

We also examined ECM gene families and several other genes whose homologs are involved in asymmetric cell divisions and spheroidal colony formation during embryogenesis in *V*. *carteri* (Supplementary Notes 1, 2). These homologs were conserved in each species of volvocine algae (Supplementary Figs. 5, 6). Although ECM gene families are highly expanded in *Eudorina* sp. and *V*. *carteri*, they were less expanded in the *A. gubernaculifera* genome than those of other volvocine species (Supplementary Fig. 7, Supplementary Data 1, 2). Therefore, whether these genes have been involved in the evolution of multicellular traits in *Astrephomene* is currently unclear.

### Germ-soma differentiation in gene expression patterns

**Figure 3:**
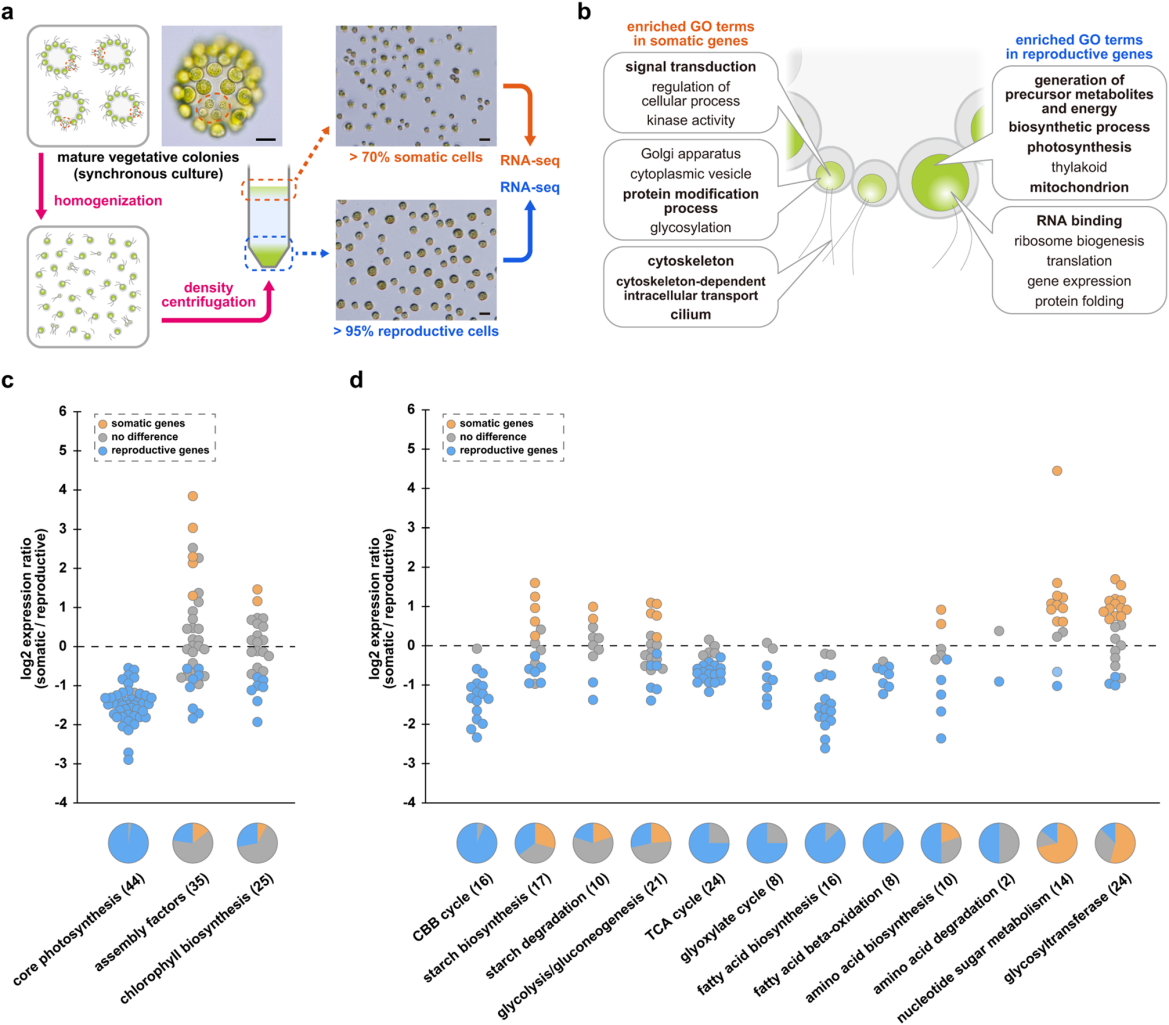
Cell-type specific RNA-seq analysis in *Astrephomene gubernaculifera*. (**a**) The workflow of isolation of somatic cells and reproductive cells. Mature vegetative colonies in synchronous culture have large reproductive cells and small somatic cells (orange dotted circles). By homogenization and density centrifugation of vegetative colonies, somatic cell and reproductive cells were separated and then subjected to RNA extraction for RNA-seq. Scale bars = 20 μm. (**b**) The representative enriched terms in somatic and reproductive genes by GO enrichment analysis. Bold texts indicate the GO terms also enriched in somatic and gonidial genes in *Volvox carteri*^32^. All other enriched GO terms were listed in Supplementary Table 3. (**c**) Gene expression ratio of photosynthesis-related genes (Supplementary Data 5). The number in brackets indicates the number of genes in each category. (**d**) Gene expression ratio of the major carbon metabolic pathways (Supplementary Data 6), nucleotide sugar metabolism and glycosyltransferases (Supplementary Data 7). The number in brackets indicates the number of genes in each category.

To examine differences in gene expression between somatic and reproductive cells in *Astrephomene*, we established a separation method for somatic and reproductive cells in *A*. *gubernaculifera* and conducted cell-type RNA-seq (Fig. 3a, Supplementary Table 2, see also Supplementary Methods). Based on differential expression analysis, we identified 2529 “somatic genes” and 2328 “reproductive genes,” which exhibited significantly higher expression in somatic or reproductive cells, respectively, among a total of 13,198 expressed genes (Supplementary Figs. 8, 9, Supplementary Data 3). GO enrichment analysis against these two groups identified many enriched terms in *A. gubernaculifera*, including “signal transduction,” “cytoskeleton-dependent intracellular transport”, and “cilium” as enriched GO terms in somatic genes, and “generation of precursor metabolites and energy” and “photosynthesis” as enriched GO terms in reproductive genes (Fig. 3b, Supplementary Table 3). These terms were common with somatic- and gonidial-enriched terms in *V*. *carteri*^32^, suggesting that germ-soma differentiation in *Astrephomene* and *Volvox* have similar features in gene expression.

We further investigated the expression patterns of *A. gubernaculifera* genes involved in respective biological functions. Among the genes involved in motility, many of the genes encoding flagella components belonged to somatic genes. In particular, most intraflagellar transport genes and Bardet-Biedel Syndrome genes were somatic genes (Supplementary Fig. 10, Supplementary Data 4). Although both somatic and reproductive cells in *Astrephomene* have flagella^33^, unlike *Volvox*, this result implies that somatic cells in *Astrephomene* are also more specialized for motility compared to reproductive cells. The most obvious difference in gene expression in *A. gubernaculifera* between somatic and reproductive cells was observed in photosynthetic genes. Almost all of the genes encoding core subunits of photosynthetic complexes on the thylakoid membranes exhibited higher expression in reproductive cells (Fig. 3c, Supplementary Fig. 11, Supplementary Data 5). On the other hand, gene expression of assembly factors for photosynthetic complexes and enzymes for chlorophyll biosynthesis did not show clear differences between somatic and reproductive cells (Fig. 3c, Supplementary Data 5). In *V*. *carteri*, almost all of the genes encoding assembly factors and enzymes for chlorophyll biosynthesis are highly expressed in gonidia^32^. Overall, both *Astrephomene* and *Volvox* are thought to have downregulation of photosynthesis in somatic cells.

Among carbon metabolism genes (Fig. 3d, Supplementary Fig. 12, Supplementary Data 6), almost all genes in the Calvin-Benson–Bassham cycle were reproductive genes in *A. gubernaculifera*. Moreover, more than half of fatty acid biosynthesis genes and half of amino acid biosynthesis genes were reproductive genes. These anabolic pathways were also upregulated in gonidia of *V*. *carteri*^32^. In addition, genes involved in acetate metabolism were reproductive in *A*. *gubernaculifera*. Acetate is mainly metabolized during the TCA cycle and glyoxylate cycle in heterotrophic algae^41^. In *A*. *gubernaculifera*, genes involved in both metabolic pathways, including enzymes for acetyl-CoA synthesis from acetate (ACK2, PAT1, and ACS1 for TCA cycle, ACS2 and ACS3 for glyoxylate cycle)^42,43^ and downstream TCA and glyoxylate cycles, were mainly upregulated in reproductive cells (Supplementary Fig. 12, Supplementary Data 6). By contrast, glyoxylate cycle genes, along with upstream fatty acid degradation, are upregulated in the somatic cells of *V*. *carteri*^32^. Differences in expression patterns of the glyoxylate cycle between *A. gubernaculifera* and *V. carteri* may reflect different trophic states: unlike *Volvox*, *Astrephomene* exhibits strong heterotrophy and requires acetate or some carboxylic acid as a carbon source for growth^36^. Thus, growth in somatic cells of *A*. *gubernaculifera* is thought to be downregulated via both phototrophic and heterotrophic pathways, whereas that of *V. carteri* somatic cells may be downregulated via only photosynthetic pathways.

**Figure 4:**
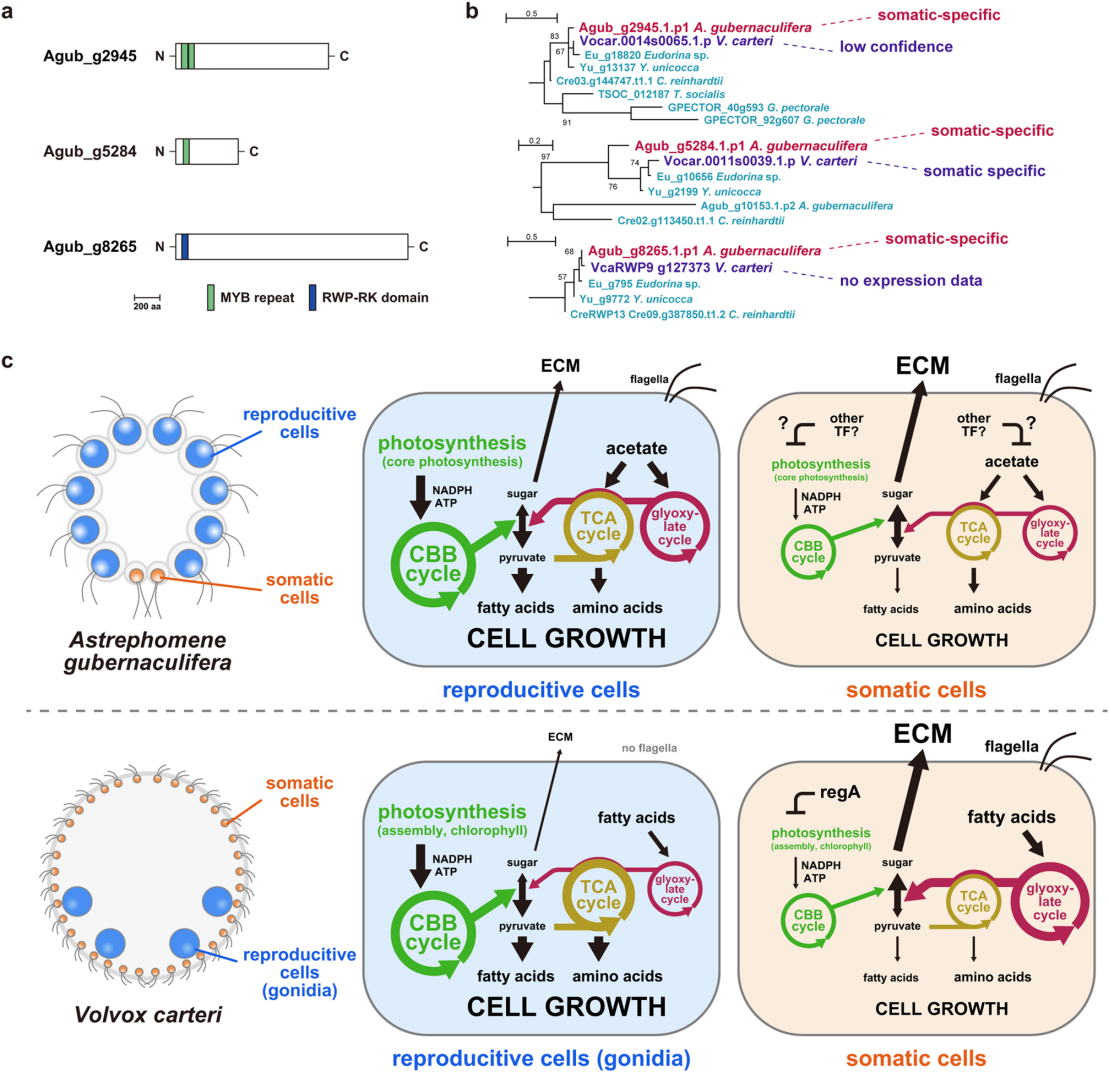
Characteristics of somatic-specific transcription factors in *Astrephomene gubernaculifera*. (**a**) The domain structure of Agub_g2945, Agub_g5284 and Agub_g8265. (**b**) Phylogenetic relationships of Agub_g2945, Agub_g5284 and Agub_g8265 among related transcription factors. The whole phylogenetic trees are shown in Supplementary Figs. 13, 14. The classification of *Volvox carteri* homologs were based on the previous study^32^, though VcaRWP9 is not found in *V*. *carteri* ver. 2.0 or 2.1 genome data and therefore there is no expression data for it. (**c**) Schematic diagrams of the comparison of germ–soma differentiation in *A. gubernaculifera* and *V. carteri*. The simplified metabolic pathways in *A*. *gubernaculifera* are based on this study, and those in *V*. *carteri* are based on a previous study^32^. The size of letters and thickness of arrows indicate gene expression levels. See also Fig. 3c,d and Supplementary Figs. 10, 11, 12.

An overview of gene expression patterns of somatic and reproductive cells in *A*. *gubernaculifera* documented in this study and comparison with those in *V*. *carteri* are summarized in Fig. 4c. In *V*. *carteri*, gonidia are larger than somatic cells at the end of embryogenesis due to the asymmetric cell divisions^44^, and the difference in cell size between cell types become much larger as gonidia grow faster than somatic cells^45,46^. This difference in cell growth between reproductive and somatic cells results from upregulation of photosynthesis in gonidia and upregulation of ECM biosynthesis in somatic cells^32^. On the other hand, the size of somatic and reproductive cells in *A*. *gubernaculifera* is almost the same at the end of embryogenesis^35^, and the difference in cell size between somatic and reproductive cells in mature colonies (Fig. 1c) may result from the different growth rates between the two cell types. The present RNA-seq analysis of *A*. *gubernaculifera* showed the upregulation of photosynthesis and acetate metabolism in reproductive cells (Fig. 3c,d, Supplementary Figs. 11, 12, Supplementary Data 5, 6), which may result in larger growth in reproductive cells than somatic cells in *A*. *gubernaculifera*. The source-sink hypothesis in Volvocales^47^, a hypothesis that somatic cells act as a source for nutrients and reproductive cells act as a sink, may not be applied to germ-soma differentiation in *Astrephomene*, since there are only two or four somatic cells in a 32- or 64-celled colony, and reproductive cells in *Astrephomene* may grow by themselves.

### Candidate genes for master regulators of germ-soma differentiation

In *V*. *carteri*, *regA* has extremely high somatic/gonidial (reproductive) expression ratios (150–1000-fold)^30,32^. To find candidate master regulator genes of germ-soma differentiation in *A*. *gubernaculifera*, we examined the transcripts of 147 somatic-specific genes that exhibited significant values of somatic/reproductive expression ratios higher than five by a statistical test (Supplementary Data 9, see also Supplementary Methods for detail). Of these, we found three genes encoding putative transcription factors: Agub_g2945, Agub_g5284, and Agub_g8265 (Fig. 4). Agub_g2945 contained two MYB repeats in a DNA binding domain (Fig. 4a) and was an R2R3-MYB transcription factor, a group that is diverged in Viridiplantae (green plants and algae)^48^ (Supplementary Fig. 13a). Agub_g5284 had one MYB repeat and was thought to be a 1R-MYB transcription factor (Fig. 4a), which belongs to a diverged group within volvocine green algae (Supplementary Fig. 13b). Though 1R-MYB genes in this group have expanded in the volvocine species, there is no tandem repeats of these genes in the volvocine genomes. On the other hand, Agub_g8265 encoded an RWP-RK transcription factor (Fig. 4a), which is included in the RKD(B) subfamily diverged in volvocine algae (Supplementary Fig. 14)^49^ and was separated from MID (sex determination)^50^ and NIT2 (nitrate signaling)^51^.

These somatic-specific transcription factors in *A*. *gubernaculifera* have putative orthologs in *V*. *carteri* and other volvocine algae (Fig. 4b, Supplementary Figs. 13, 14). Because the homolog of Agub_g2945, Vocar.0014s0065, exhibits low expression in both somatic cells and gonidia in *V*. *carteri*^32^, the somatic-specific expression of Agub_g2945 may only have evolved in the ancestor of *Astrephomene*. On the other hand, because both Agub_g5284 and its homolog Vocar.0011s0039 exhibit somatic-specific expression^32^, these homologs may represent convergent evolution of upregulated gene expression in somatic cells between *Astrephomene* and *Volvox*. Agub_8265 is the homolog of VcaRWP9^49^, which is absent from the most recent gene models of the *V*. *carteri* genome (ver. 2.0 and 2.1).

A previous study showed that *regA* as wells as several other VARL genes have high expression in somatic cells in *V. carteri*^32^. Among six VARL genes in *A*. *gubernaculifera* (Supplementary Note 3, Supplementary Data 8), the *rlsH* homolog had high somatic/reproductive expression ratio, though it was only detected as somatic-biased gene (Supplementary Methods). On the other hand, *V*. *carteri rlsH* expression was reported as somatic-specific^32^. Thus, the *rlsH* homologs in *A*. *gubernaculifera* and *V*. *carteri* may have similar function in somatic cells, which is currently unknown. As *A*. *gubernaculifera* does not have a *regA*-like gene (Fig. 2), the *rlsH* homolog also might be involved in the differentiation of somatic cells in *A*. *gubernaculifera*.

Although the functions of the three somatic-specific transcription factors in *A*. *gubernaculifera* found in this study and their closely related homologs in other volvocine algae (Fig. 4) are currently unknown, these genes warrant further investigation as candidates of master regulators of germ-soma differentiation in *A*. *gubernaculifera*. In addition to these transcription factors and the *rlsH* homolog, it is possible that the post-transcriptional regulation of other transcription factors may be involved in the cell differentiation, which would be discovered by further studies on *Astrephomene*. The difference in such regulator genes between *A*. *gubernaculifera* and *V*. *carteri* may also affect differences in the downregulation of metabolic pathways (especially in acetate metabolism) to repress the growth of somatic cells (Fig. 4c).

## Conclusion

Convergent evolution, the independent evolution of similar traits in two or more different groups, is a crucial topic in evolutionary biology. Recent studies with genetic and genomic tools have revealed that convergent evolution has occurred at various hierarchical levels even for the similar phenotype: in mutations, genes, gene functions, networks or pathways^52,53^. Germ-soma differentiation is a pivotal trait in convergent evolution, as it has occurred in many multicellular lineages and contributed to the evolution of multicellular complexity^3,22^. However, it has been unsolved at what level the convergence of germ-soma occurred. Here, we documented a hierarchy in the convergent evolution of germ-soma differentiation by focusing on *Volvox* and *Astrephomene* (Fig. 1). Through de novo genome sequencing and RNA-seq analyses of *A*. *gubernaculifera*, we revealed differences in the regulation of germ-soma differentiation at the genetic level. While the master regulator gene for germ-soma differentiation in *V*. *carteri* is *regA*, we demonstrated that there is no *regA* or no *reg* cluster gene in the *Astrephomene* genome (Fig. 2). However, we discovered three somatic-specific transcription factors that are candidate genes for regulating germ-soma differentiation in *Astrephomene.* Although *regA* belongs to the VARL gene family with the SAND domain, these three *Astrephomene* candidate genes belong to either the MYB or RWP-RK gene family (Fig. 4). Nevertheless, the present and previous cell-type RNA-seq analyses of *Astrephomene* and *Volvox* have demonstrated that these two multicellular organisms share a common overall pattern of two cell types in gene expression at the metabolic pathway level: reproductive cells are specialized for cell growth and reproduction, while somatic cells are specialized for motility and ECM biosynthesis instead of cell growth (Fig. 3). Thus, different transcription factors in the ancestors of *Astrephomene* and *Volvox* may have been independently co-opted for a similar function in the regulation of gene expression in differentiated cells, which would underlie the convergent evolution of germ-soma differentiation within the volvocine green algae. This contrasts with the recent studies which have reported that the repeated evolution of the same transcription factor underlies phenotypic convergence, such as parallel duplication of Zn-cluster TFs associated with multiple emergences of unicellular yeast-like fungi^54^.

The present study suggested that the convergent evolution of germ-soma differentiation in *Volvox* and *Astrephomene* is based on evolution of different transcription factors that control similar gene expression patterns in the different linages (Fig. 4c). This evolution may involve various changes at many levels: expression and/or regulation of the transcription factors themselves, *cis*-regulatory elements they bind, target genes and/or gene circuits having the *cis*-regulatory elements, etc. Thus, the convergent evolution of germ-soma differentiation in *Volvox* and *Astrephomene* may be much more complicated than previous, well-known examples of convergent evolution in animals, such as convergence of pigmentation by the mutation in the same pathway (but different genes or different gene function) in mice^55^ and lizards^56^. Further study on the convergent evolution of germ-soma differentiation in *Astrephomene* and *Volvox* would require the new approach to finding the evolutionary changes, such as comparative ChIP-seq analysis between volvocine species, after the identification of the master regulator for somatic cell differentiation in *Astrephomene*. The convergent evolution of germ-soma differentiation in volvocine algae would be a simple, suitable model for understanding how different transcription factors have been co-opted for the evolution of a similar phenotype.

## Methods

### Strain and culture conditions

*Astrephomene gubernaculifera* strain NIES-4017 (from the Microbial Culture Collection at the National Institute for Environmental Studies [NIES]; http://mcc.nies.go.jp/^35,57^) was used throughout this study. The culture was maintained in 10 mL of VTAC medium^57,58^ in screw-capped tubes (18 × 150 mm) at 25 °C on a 12-h light/12-h dark schedule under cool-white fluorescent lamps at an intensity of 50–90 μmol·m^−2^·s^−1^. For experimental analyses, synchronous cultures were established as described previously^35^ (Fig. 1f, Supplementary Methods).

### De novo genome sequencing and assembly

For extraction of genomic DNA, young colonies just after embryogenesis in synchronous cultures of *A*. *gubernaculifera* (ZT 15 in Fig. 1f) were used. Genomic DNA from cells was prepared based on methods described previously^59^. A 30-kb SMRTbell library was constructed using a SMRTbell Express Template Prep Kit 2.0 (Pacific Biosciences, CA, USA) according to the manufacturer’s protocol. The sequencing library was size-selected using the BluePippin system (Sage Science, MA, USA) with a minimum fragment length cutoff of 30 kbp. Three SMRT cells (1M v3 LR) were run on the PacBio Sequel System with Binding Kit3.0/Sequencing Kit3.0. This reaction generated 2.6 M subreads (total bases: 39.5 Gb). Sequencing coverage was approximately 380 × based on the estimated genome size. Illumina paired-end libraries (average insert sizes of 600 bp) were constructed from the same genomic DNA using a TruSeq DNA Sample Prep Kit (Illumina, CA, USA) according to the manufacturer’s instructions. This library was sequenced on an Illumina HiSeq 2500 sequencer (71.4 M reads with 250-bp read length). Total bases and sequencing coverage were 17.8 Gb (172 ×).

For *A*. *gubernaculifera* nuclear genome assembly, PacBio subreads were first assembled de novo using Falcon v0.7, Falcon-unzip v0.4^60^, and SMRT Link v6.0.0.47841 (Pacific Biosciences). The Illumina data were then mapped against the PacBio assembly sequence, and assemblies were corrected using Pilon v1.22^61^. Contigs with < 45% GC (putative organelle genome) were excluded from a set of the nuclear genome sequence. The quality of genome assembly was verified using BUSCO ver.5.0.0^37^ with the chlorophyta_odb10 dataset (1519 BUSCOs), which indicated that 97.1% complete genes in the reference dataset were present in the current genome assembly (C: 97.1% S: 96.6% D: 0.5% F: 0.9% M: 2.0%). Plastid and mitochondrial genomes were also assembled and annotated, respectively (Supplementary Methods).

### Prediction of gene models of *Astrephomene gubernaculifera* using RNA-seq data

For extraction of total RNA for annotation of the nuclear genome of *A*. *gubernaculifera*, young colonies and embryos in synchronous culture (ZT 23 and ZT 11 in Fig. 1f) were used. RNA was extracted using TRIzol reagent (Thermo Fisher Scientific, MA, USA) according to the manufacturer’s instructions. Illumina RNA-seq libraries were constructed using a TruSeq Stranded mRNA Library Prep Kit (Illumina). These libraries were sequenced on an Illumina HiSeq 2500 sequencer (Illumina). 22.6 M and 24.2 M paired-end reads (100 bp × 2) were generated for young colonies and embryos, respectively. The sequenced reads from two samples were merged and aligned to the *A*. *gubernaculifera* genome assembly described above using HISAT2 version 2.1.0^62^. 89.0% of read pairs were uniquely mapped to the genome. The mapped reads were assembled using StringTie v2.0.4^63^ and the coding regions in assemblies generated by StringTie were identified using TransDecoder v5.5.0 (https://github.com/TransDecoder/TransDecoder) for predicted gene models.

### Identification and analysis of VARL genes and other genes related to multicellular traits

The VARL genes and gene synteny near the *RLS1*/*rlsD* ortholog in *A*. *gubernaculifera* were identified based on a BLASTP search and phylogenetic analysis (Supplementary Methods). Homologs of genes involved in embryogenesis in *V*. *carteri* were searched among genome data of volvocine species (Supplementary Table 4) primarily using a BLASTP search (Supplementary Methods). The amino acid sequences of InvA orthologs in several volvocine species were determined by PCR and sequencing the PCR products using newly designed primers (Supplementary Table 5, see Supplementary Methods for detail). ECM gene families that are expanded in *V*. *carteri* were searched among volvocine species using protein model-based search methods (Supplementary Methods) based on a previous study^9^.

### Cell-type RNA-seq analysis

The separation of somatic and reproductive cells of *A*. *gubernaculifera*, RNA-seq analysis using both cell samples and differential expression analysis were performed as shown in Fig. 3a and described in detail in Supplementary Methods. For characterization of gene expression patterns in somatic and reproductive cells, we conducted Gene Ontology (GO) enrichment analysis of differentially expressed genes (Supplementary Methods) and analyses of gene expression involved in motility, photosynthesis, carbon metabolism, and multicellularity (Supplementary Methods, Supplementary Data 4–8). The latter were mainly based on BLASTP search using homologs in *C*. *reinhardtii*^43^ and *V*. *carteri*^32^ (Supplementary Methods) and phylogenetic analysis of LHCII and LHCI genes (Supplementary Fig. 15).

### Identification and analysis of somatic- and reproductive-specific genes

The characteristics of proteins encoded in all somatic- and reproductive-specific genes described above were deduced based on a BLASTP search against the NCBI nonredundant database (E-value < 1e−10) using DIAMOND v2.0.2.140^64^ and a domain search against all Pfam-A models^65^ using “hmmscan” (E-value < 1e−5) in HMMER. The putative hydroxyproline-rich domains, which contain prolines at a high ratio, were difficult to detect by these searches and were instead found by checking sequences manually. Among somatic-specific genes, putative MYB transcription factors, Agub_g2945 and Agub_g5284, and a putative RWP-RK transcription factor, Agub_g8265, were subjected to phylogenetic analysis (Supplementary Methods).

### Statistics and reproducibility

For cell-type RNA-seq analysis, we isolated three somatic and reproductive cell samples from different flasks containing *A*. *gubernaculifera* culture, respectively, and treated as biological replicates (Supplementary Table 2). The differential expression analysis was based on Wald test between the three somatic and three reproductive replicates using DESeq2^66^. GO enrichment analysis was based on two-sided Fisher’s exact test for the detected differential expressed genes (somatic and reproductive genes) using Blast2GO^67^. For both tests, statistical significance was defined as a false discovery rate (FDR) < 0.05. See Supplementary Methods for detail.

## Supplementary Information

Supplementary Information 1.

Supplementary Information 2.

Supplementary Information 3.

Supplementary Information 4.

Supplementary Information 5.

Supplementary Information 6.

Supplementary Information 7.

Supplementary Information 8.

Supplementary Information 9.

Supplementary Information 10.

Supplementary Information 11.

Supplementary Information 12.

## Data Availability

The nuclear genome assembly with annotated gene models of *A*. *gubernaculifera* strain NIES-4017 has been deposited to DDBJ/EMBL/GenBank (BioProject accession number: PRJDB10253; BioSample accession number: SAMD00236769; WGS accession numbers: BMAR01000001–BMAR01000206). The plastid and mitochondrial genome assemblies with annotated gene models of *A*. *gubernaculifera* strain NIES-4017 have been deposited to DDBJ/EMBL/GenBank (accession number: LC523292 [plastid], AP024562 [mitochondria]). The raw read sequence data from cell-type RNA-seq analysis also has been deposited to DDBJ/EMBL/GenBank (BioProject accession number: PRJDB10862; BioSample accession numbers: SAMD00260684–SAMD00260689; DRA accession number: DRA011195). The alignment file and dataset partitioning and best-fitted substitution models used for phylogenomic analysis of volvocine species is Supplementary Data 10 and 11 for this article, respectively. All the other sequence alignments used for phylogenetic analyses have been deposited in TreeBASE (https://treebase.org/treebase-web/home; study ID: 27376). All the other data generated in this study are included in this published article and Supplementary information files.

## References

[1] Smith JM, Száthmary E (1995). The Major Transitions in Evolution.

[2] Queller DC (2000). Relatedness and the fraternal major transitions. Philos. Trans. R. Soc. Lond. B Biol. Sci..

[3] Grosberg RK, Strathmann RR (2007). The evolution of multicellularity: A minor major transition?. Annu. Rev. Ecol. Evol. Syst..

[4] Rensing SA (2016). (Why) Does evolution favour embryogenesis?. Trends Plant Sci..

[5] Kirk DL (2005). A twelve-step program for evolving multicellularity and a division of labor. BioEssays.

[6] Herron MD, Hackett JD, Aylward FO, Michod RE (2009). Triassic origin and early radiation of multicellular volvocine algae. Proc. Natl. Acad. Sci. U.S.A..

[7] Merchant SS (2007). The *Chlamydomonas* genome reveals the evolution of key animal and plant functions. Science.

[8] Prochnik SE (2010). Genomic analysis of organismal complexity in the multicellular green alga *Volvox carteri*. Science.

[9] Hanschen ER (2016). The *Gonium pectorale* genome demonstrates co-option of cell cycle regulation during the evolution of multicellularity. Nat. Commun..

[10] Featherston J (2018). The 4-celled *Tetrabaena socialis* nuclear genome reveals the essential components for genetic control of cell number at the origin of multicellularity in the volvocine lineage. Mol. Biol. Evol..

[11] Hamaji T (2018). Anisogamy evolved with a reduced sex-determining region in volvocine green algae. Commun. Biol..

[12] Kindle KL, Schnell RA, Fernández E, Lefebvre PA (1989). Stable nuclear transformation of *Chlamydomonas* using the *Chlamydomonas* gene for nitrate reductase. J. Cell Biol..

[13] Schiedlmeier B (1994). Nuclear transformation of *Volvox carteri*. Proc. Natl. Acad. Sci. U.S.A..

[14] Lerche K, Hallmann A (2009). Stable nuclear transformation of *Gonium pectorale*. BMC Biotechnol..

[15] Lerche K, Hallmann A (2013). Stable nuclear transformation of *Eudorina elegans*. BMC Biotechnol..

[16] Lerche K, Hallmann A (2014). Stable nuclear transformation of *Pandorina morum*. BMC Biotechnol..

[17] Weismannn A (1892). Das Keimplasma; eine Theorie der Vererbung.

[18] Koufopanou V (1994). The evolution of soma in the Volvocales. Am. Nat..

[19] Solari CA, Kessler JO, Michod RE (2006). A hydrodynamics approach to the evolution of multicellularity: Flagellar motility and germ-soma differentiation in Volvocalean green algae. Am. Nat..

[20] Solari CA, Ganguly S, Kessler JO, Michod RE, Goldstein RE (2006). Multicellularity and the functional interdependence of motility and molecular transport. Proc. Natl. Acad. Sci. U.S.A..

[21] Michod RE, Roze D (2001). Cooperation and conflict in the evolution of multicellularity. Heredity.

[22] Rose CJ (2020). Germ lines and extended selection during the evolutionary transition to multicellularity. J. Exp. Zool. B Mol. Dev. Evol..

[23] Michod RE, Viossat Y, Solari CA, Hurand M, Nedelcu AM (2006). Life-history evolution and the origin of multicellularity. J. Theor. Biol..

[24] Herron MD, Michod RE (2008). Evolution of complexity in the volvocine algae: Transitions in individuality through Darwin’s eye. Evolution.

[25] Nozaki H, Ito M (1994). Phylogenetic relationships within the colonial Volvocales (Chlorophyta) inferred from cladistic analysis based on morphological data. J. Phycol..

[26] Kirk MM (1999). *regA*, a *Volvox* gene that plays a central role in germ-soma differentiation, encodes a novel regulatory protein. Development.

[27] Duncan L (2007). The *VARL* gene family and the evolutionary origins of the master cell-type regulatory gene, *regA*, in *Volvox carteri*. J. Mol. Evol..

[28] Hanschen ER, Ferris PJ, Michod RE (2014). Early evolution of the genetic basis for soma in the Volvocaceae: Evolution of the genetic basis for soma. Evolution.

[29] Grochau-Wright ZI (2017). Genetic basis for soma is present in undifferentiated volvocine green algae. J. Evol. Biol..

[30] Nematollahi G, Kianianmomeni A, Hallmann A (2006). Quantitative analysis of cell-type specific gene expression in the green alga *Volvox carteri*. BMC Genomics.

[31] Klein B, Wibberg D, Hallmann A (2017). Whole transcriptome RNA-Seq analysis reveals extensive cell type-specific compartmentalization in *Volvox carteri*. BMC Biol..

[32] Matt GY, Umen JG (2018). Cell-type transcriptomes of the multicellular green alga *Volvox carteri* yield insights into the evolutionary origins of germ and somatic differentiation programs. Genes Genomes Genetics.

[33] Pocock MA (1954). Two multicellular motile green algae, *Volvulina* Playfair and *Astrephomene*, a new genus. Trans. R. Soc. South Afr..

[34] Nozaki H (1983). Morphology and taxonomy of two species of *Astrephomene* (Chlorophyta, Volvocales) in Japan. J. Jpn. Bot..

[35] Yamashita S (2016). Alternative evolution of a spheroidal colony in volvocine algae: Developmental analysis of embryogenesis in *Astrephomene* (Volvocales, Chlorophyta). BMC Evol. Biol..

[36] Brooks AE (1972). The physiology of *Astrephomene gubernaculifera*. J. Protozool..

[37] Simão FA, Waterhouse RM, Ioannidis P, Kriventseva EV, Zdobnov EM (2015). BUSCO: Assessing genome assembly and annotation completeness with single-copy orthologs. Bioinformatics.

[38] Lindsey CR, Rosenzweig F, Herron MD (2021). Phylotranscriptomics points to multiple independent origins of multicellularity and cellular differentiation in the volvocine algae. BMC Biol..

[39] Nedelcu AM, Michod RE (2006). The evolutionary origin of an altruistic gene. Mol. Biol. Evol..

[40] Nedelcu AM (2009). Environmentally induced responses co-opted for reproductive altruism. Biol. Lett..

[41] Perez-Garcia O, Escalante FME, Bashan LE, de-Bashan Y (2011). Heterotrophic cultures of microalgae: Metabolism and potential products. Water Res..

[42] Yang W (2014). Alternative acetate production pathways in *Chlamydomonas reinhardtii* during dark anoxia and the dominant role of chloroplasts in fermentative acetate production. Plant Cell.

[43] Zones JM, Blaby IK, Merchant SS, Umen JG (2015). High-resolution profiling of a synchronized diurnal transcriptome from *Chlamydomonas reinhardtii* reveals continuous cell and metabolic differentiation. Plant Cell.

[44] Green K, Kirk DL (1981). Cleavage patterns, cell lineages, and development of a cytoplasmic bridge system in *Volvox* embryos. J. Cell Biol..

[45] Kirk MM, Ransick A, McRae SE, Kirk DL (1993). The relationship between cell size and cell fate in *Volvox carteri*. J. Cell Biol..

[46] Kirk DL (1998). Volvox: Molecular-Genetic Origins of Multicellularity and Cellular Differentiation.

[47] Bell, G. The origin and early evolution of germ cells as illustrated by the Volvocales. in *The Origin and Evolution of Sex* 221–256 (Alan R. Liss, 1985).

[48] Du H (2015). The evolutionary history of R2R3-MYB proteins across 50 eukaryotes: New insights into subfamily classification and expansion. Sci. Rep..

[49] Chardin C, Girin T, Roudier F, Meyer C, Krapp A (2014). The plant RWP-RK transcription factors: Key regulators of nitrogen responses and of gametophyte development. J. Exp. Bot..

[50] Ferris PJ, Goodenough UW (1997). Mating type in chlamydomonas is specified by *mid*, the minus-dominance gene. Genetics.

[51] Camargo A (2007). Nitrate signaling by the regulatory gene *NIT2* in *Chlamydomonas*. Plant Cell.

[52] Manceau M, Domingues VS, Linnen CR, Rosenblum EB, Hoekstra HE (2010). Convergence in pigmentation at multiple levels: Mutations, genes and function. Philos. Trans. R. Soc. B Biol. Sci..

[53] Rosenblum EB, Parent CE, Brandt EE (2014). The molecular basis of phenotypic convergence. Annu. Rev. Ecol. Evol. Syst..

[54] Nagy LG (2014). Latent homology and convergent regulatory evolution underlies the repeated emergence of yeasts. Nat. Commun..

[55] Steiner CC, Weber JN, Hoekstra HE (2007). Adaptive variation in beach mice produced by two interacting pigmentation genes. PLoS Biol..

[56] Rosenblum EB, Römpler H, Schöneberg T, Hoekstra HE (2010). Molecular and functional basis of phenotypic convergence in white lizards at White Sands. Proc. Natl. Acad. Sci. U.S.A..

[57] Kawachi, M. *et al.* MCC-NIES list of strains, 9th edition, microbial culture collection at National Institute for Environmental Studies, Tsukuba, Japan. https://mcc.nies.go.jp/download/list9th_e.pdf (2013).

[58] Nozaki H, Kuroiwa H, Mita T, Kuroiwa T (1989). *Pleodorina**japonica* sp. nov. (Volvocales, Chlorophyta) with bacteria-like endosymbionts. Phycologia.

[59] Miller SM, Schmitt R, Kirk DL (1993). Jordan, an active *Volvox* transposable element similar to higher plant transposons. Plant Cell.

[60] Chin C-S (2016). Phased diploid genome assembly with single-molecule real-time sequencing. Nat. Methods.

[61] Walker BJ (2014). Pilon: An integrated tool for comprehensive microbial variant detection and genome assembly improvement. PLoS One.

[62] Kim D, Paggi JM, Park C, Bennett C, Salzberg SL (2019). Graph-based genome alignment and genotyping with HISAT2 and HISAT-genotype. Nat. Biotechnol..

[63] Kovaka S (2019). Transcriptome assembly from long-read RNA-seq alignments with StringTie2. Genome Biol..

[64] Buchfink B, Xie C, Huson DH (2015). Fast and sensitive protein alignment using DIAMOND. Nat. Methods.

[65] El-Gebali S (2019). The Pfam protein families database in 2019. Nucleic Acids Res..

[66] Love MI, Huber W, Anders S (2014). Moderated estimation of fold change and dispersion for RNA-seq data with DESeq2. Genome Biol..

[67] Conesa A (2005). Blast2GO: A universal tool for annotation, visualization and analysis in functional genomics research. Bioinformatics.

