# Supplementary material for: Genome sequencing of the multicellular alga Astrephomene provides insights into convergent evolution of germ-soma differentiation
Source: Sci Rep. 2021 Nov 22;11:22231. doi: 10.1038/s41598-021-01521-x (PMC8608804; doi:10.1038/s41598-021-01521-x)
Supplement: Supplementary file 12 — Supplementary Information 12. [file 41598_2021_1521_MOESM12_ESM.pdf]

Supplementary Information for

Genome sequencing of the multicellular alga *Astrephomene* provides insights into convergent evolution of germ-soma differentiation

Shota Yamashita, Kayoko Yamamoto, Ryo Matsuzaki, Shigekatsu Suzuki, Haruyo Yamaguchi, Shunsuke Hirooka, Yohei Minakuchi, Shin-ya Miyagishima, Masanobu Kawachi, Atsushi Toyoda and Hisayoshi Nozaki*

*Corresponding Author: Hisayoshi Nozaki
Email: nozaki@bs.s.u-tokyo.ac.jp

Supplementary Notes

Supplementary Note 1. Homologs of genes involved in embryogenesis in *Volvox carteri*

In embryogenesis in *Volvox carteri*, a part of cells of the embryo divide asymmetrically, which results in the formation of large cells (gonidial initials)[1]. As the cell sheet of an embryo after successive cell divisions is inside-out with respect to the adult configuration, the embryo then performs inversion to form the adult configuration[2,3]. Three genes involved in inversion, *InvA*[4], *InvB* and *InvC*[5,6], and two genes involved in asymmetric cell division, *GlsA*[7] and *Hsp70A*[8], have been identified so far. On the other hand, the embryo in *Astrephomene* forms the proper configuration directly by the outward rotation of daughter protoplasts during successive cell divisions and does not perform inversion[9]. Distinct asymmetric cell division also does not occur to form somatic and reproductive cells in *Astrephomene*[9].

Orthologs of *V. carteri* genes which are involved in inversion (*InvA*, *InvB* and *InvC*) and asymmetric cell divisions (*GlsA* and *Hsp70A*) were searched in the present genome data of *A. gubernaculifera* as well as other volvocine algae (Supplementary Table 4). *A. gubernaculifera* had an ortholog of each of the five genes. Orthologs of these genes were also found in all other species, except for *InvC* ortholog in *Gonium pectorale* and *GlsA* ortholog in *Tetrabaena socialis* (Supplementary Fig. 5). Amino acid sequences of orthologs of each gene were conserved (InvA: >81%, InvB: >71%, InvC: >55%, GlsA: >75%, Hsp70A: >95%). Each ortholog also has similar exon–intron structure among volvocine species (Supplementary Fig. 6). As *Astrephomene* embryo does not undergo inversion or asymmetric cell divisions, these orthologous genes in *Astrephomene* might have different functions from *Volvox* in cellular level.

Supplementary Note 2. ECM genes

The components of expanded ECM in *V. carteri* have been identified so far and most of them are revealed to be glycoproteins with hydroxyproline-rich domains (hydroxyproline-rich glycoproteins,

HRGPs)[10]. Among them, numbers of matrix metalloproteases (MMPs) and pherophorins are remarkably increased in *V. carteri* genome compared with those in *Chlamydomonas reinhardtii*, *T. socialis* or *G. pectorale* genome[11–13]. *Astrephomene* also has expanded ECM, though the expanded regions of ECM are different from Volvocaceae in their structure[14,15]. The expanded ECM in *Astrephomene* was estimated to have been acquired independently of *Volvox* and Volvocaceae[16,17], though the present phylogenomic analysis (Supplementary Fig. 4) and a recent phylotranscriptomics study[18] do not support this independency.

Our protein model-based search of ECM genes (see Supplementary Methods) revealed that *A. gubernaculifera* had only 20 MMP genes and 54 pherophorin genes, which are both the smallest numbers among volvocine algae (Supplementary Fig. 7, Supplementary Data 1, 2). It is in contrast with the high number of these genes in Volvocaceae. This result suggests that the expansion of ECM in *Astrephomene* was not accompanied by the expansion of MMPs or pherophorins in contrast to Volvocaceae. From the comparison between *A. gubernaculifera* and *Yamagishiella unicocca*, the number of these ECM genes is thought to be involved in the difference in complexity of ECM structures[14,15,19] , rather than the volume of ECM. The number of these genes in anisogamous *Eudorina* sp. and oogamous *V. carteri* were greater than those in isogamous *Y. unicocca*. As some of pherophorin genes have reported to be involved in the signal amplification of sex inducer[20,21], the expansion of these ECM gene families may be involved in the evolution of sexual reproduction, the transition from isogamy to anisogamy and oogamy[22,23], in addition to the complexity of ECM structures.

Supplementary Note 3. Cell-type expression of genes related to multicellularity in *Astrephomene gubernaculifera*

In *Volvox carteri*, the somatic cell-differentiating master gene (*regA*) belongs to the VARL gene family and has extremely high somatic/gonidial (reproductive) expression ratios (150-1,000-fold)[24,25].

Therefore, we examined expression of VARL genes in *A. gubernaculifera* (Supplementary Data 8). Agub_g12775 (*RLS1*/*rlsD* ortholog), Agub_g8757 (*RLS10*/*rlsL* related) and Agub_g12169 (*RLS5, 6, 9*/*rlsH* related) were somatic genes, while Agub_g12876 (*rlsF* related) was reproductive gene. Agub_g12169 (*RLS5, 6, 9*/*rlsH* related) had the highest somatic/reproductive expression ratio (8.6-fold). In *V. carteri*, some of VARL genes were upregulated in somatic cells and *rlsH* expression was somatic-specific[24]. Thus, the *rlsH* homologs in *A. gubernaculifera* and *V. carteri* might have similar function, which is currently unknown.

Moreover, expression the other genes related in multicellular traits was also examined (Supplementary Data 8). Among orthologous genes involved in embryogenesis in *V. carteri*, Agub_g14864 (*InvB* ortholog) and Agub_g8128 (*GlsA* ortholog) were reproductive genes. As the reproductive cells are going to undergo embryogenesis while somatic cells are not, these genes might be involved in embryogenesis also in *Astrephomene*.

In terms of the ECM components, the half of MMP genes also belonged to somatic genes, while the expression of pherophorin genes varied (Supplementary Data 8). Moreover, majority of genes involved in biosynthesis of nucleotide sugars and glycosyltransferase genes, which are used in glycosylation of ECM glycoprotein in volvocine algae[10], belonged to somatic genes in *A. gubernaculifera* (Fig. 3d, Supplementary Fig. 12, Supplementary Data 7), which is also the same as *V. carteri*[24]. In addition, many of somatic-specific genes have proline-lich regions which are similar to those of hydroxyproline-rich glycoproteins constituting ECM (Supplementary Data 9). These results might indicate the enhancement of ECM biosynthesis in somatic cells in *A. gubernaculifera*. In *V. carteri*, most of ECM of a colony is produced by somatic cells as the sugar sink[24]. In *Astrephomene*, however, the somatic cells constitute only a small region of posterior pole in a colony and do not seem to contribute to production of whole ECM of the colony. Thus, the upregulation of genes involved in ECM biosynthesis in somatic cells of *Astrephomene* might reflect the larger proportion of ECM volume to cell volume in small somatic cells (Fig. 1c).

Supplementary Methods

Synchronous culture of *Astrephomene gubernaculifera*

The synchronous culture of *A. gubernaculifera* NIES-4017[9,26] was established as previously reported[9]. The culture were grown in silicon-capped 500 mL Erlenmeyer flasks containing 250 mL VTAC medium with aeration, at 32°C on a 16-h light/8-h dark schedule under cool-white fluorescent lamps at an intensity of 140–180 $\mu mol \cdot m^{-2} \cdot s^{-1}$. The inoculation of culture was conducted every 24 h, just before the onset of the dark period (ZT 15.5 in Fig. 1f), by transferring 4 mL of culture into new 250 mL VTAC medium in silicon-capped Erlenmeyer flasks. In this condition, the asexual life cycle was synchronized and completed in approximately 24 h, and the culture was highly synchronized with the light–dark cycle. Almost all reproductive cells (> 95%) of *A. gubernaculifera* initiated embryogenesis approximately 9–10 h after the onset of the light period (Fig. 1f).

Assembly and annotation of organelle genomes in *Astrephomene gubernaculifera*

The plastid and mitochondrial genomes of *A. gubernaculifera* were constructed as follows. First, Illumina paired-end and mate pair reads were assembled using Platanus v1.2.4[27] with the default parameters. Second, scaffold sequences derived from plastid and mitochondrial genomes were selected by mapping to the *V. africanus* plastid genome (accession number: NC_039755.1)[28] and mitochondrial genome (unpublished) using NCBI BLAST v2.2.9[29] with the parameters of "-p tblastx -F F -e 1e-10". Third, PacBio subreads were mapped to the selected scaffold sequences using BLASR v.5.1[30], and the mapped subreads for the plastid and mitochondrial genomes were assembled using Canu v2.1[31] and MaSuRCA v3.2.8[32], respectively, with the default parameters. Finally, the lllumina assembly sequences were used to close the gaps in the PacBio assemblies of both genomes and error correction was performed using pilon v1.22[33]. The genes on both plastid and mitochondrial genome sequences were annotated using GeSeq[34] on Chlorobox (https://chlorobox.mpimp-golm.mpg.de/index.html) with available plastid and mitochondrial

genomes of volvocine species as references.

Genome wide gene comparison and dN/dS analysis

The comparison of the orthogroup contents in genome data of *A. gubernaculifera*, *V. carteri*, *G. pectorale* and *C. reinhardtii* was conducted gene clustering analysis against available gene models of the four species (Supplementary Table 4) using OrthoFinder v2.5.4[35]. For 6,110 orthogroups which were shared in the four species as a single copy orthologs ("1:1:1:1" orthologs in Supplementary Fig 2b), were then subjected to dN/dS analysis, based on methods in a previous study[12]. The amino acid sequences of four orthologs in 6,110 orthogroups were aligned using MUSCLE v3.8.31[36]. 19 orthogroups in which the length of available alignment shared by all four orthologs is less than 30 amino acids were removed from further analysis. DNA sequences of four orthologs in 6,091 orthogroups were aligned according to the alignment of amino acid sequences using PAL2NAL v14[37] and pairwise dN, dS and dN/dS were calculated using codeml in PAML package[38].

Phylogenetic analyses using nuclear genome-encoded proteins

We performed phylogenetic analyses using 371 nuclear genome-encoded proteins. For the database, nuclear genomes of *Yamagishiella unicocca* (accession number: BDSL00000000) and *Eudorina* sp. NIES-3984 (accession number: BDSI00000000)[23] were annotated and RNA-seqs of *Vitreochlamys ordinata* NIES-882 (accession number: SRR13719284), *V. aulata* NIES-878 (accession number: SRR13719285), and *V. nekrassovii* SAG 11-10 (accession number: SRR13719243)[18] were assembled in this study. Repeat sequences of the genomes of *Y. unicocca* and *Eudorina* sp. were masked using RepeatModeler v. 2.0.1[39] and RepeatMaker v. 4.1.1 (https://www.repeatmasker.org/). Gene models were predicted using funannotate pipeline v. 1.8.7 (https://github.com/nextgenusfs/funannotate). The RNA-seqs were assembled using Trinity v. 2.11.0[40] and long ORFs were extracted using TransDecoder v. 5.5.0 (https://github.com/TransDecoder/TransDecoder). The database included 14

genomes or transcriptomes. The highly conserved orthologs were searched using the reciprocal best-hit analyses using blastp with cut-off: similarity >70% and HSP coverage >50%. Completely redundant proteins were removed and the proteins of *C. reinhardtii* were used as a reference. The 371 orthologous proteins were shared by all of the species. The amino acid sequences were aligned using MAFFT v. 7.487 with an auto option[41]. The alignments were automatically trimmed using trimAl v. 1.4.1 with the option, automated1[42]. The trimmed dataset contained 140,917 amino acids. The model test was performed using ModelTest-NG v. 0.1.6[43]. Maximum likelihood analysis was performed with 200 bootstrap replicates[44] using RAxML-NG v. 1.0.3[45]. For Bayesian analysis, MrBayes v3.2.7a[46] was used with WAG + GAMMA + I substitutional model, which was tested using ModelTest-NG. Bayesian inference consisted of 1,000,000 generations with sampling at every 1,000 generations using the four Metropolis-coupled Markov chain Monte Carlo simulations. Two separate runs were performed, and the convergence was assessed by the average standard deviation of split frequencies (ASDSF) falling below 0.01. Bayesian posterior probabilities (BPP) were calculated from the majority rule consensus of the trees sampled after the initial 250 burn-in trees.

Identification and analysis of VARL genes

The master regulator of somatic cells in *V. carteri*, *regA*, encodes a transcription factor with a DNA-binding SAND-like domain (called VARL domain, volvocine algae *regA*-like)[47]. *regA* and other previously reported VARL genes (genes encoding putative transcription factors with VARL domain) in *V. carteri*, *Y. unicocca*, *G. pectorale* and *C. reinhardtii*[12,48,49] were used as queries in BLASTP search (E-value < 1e-10) against gene models of *A. gubernaculifera* described above. The VARL domain of each gene, the N-terminal extension and core VARL domain structure[48], was identified based on alignment with VARL genes in volvocine algae[49]. The 8 VARL genes in *T. socialis* corresponding to those reported in a previous study[13] and their VARL domains were also identified by the same method. The amino acid sequences of VARL domains from *A. gubernaculifera*,

T. socialis and other volvocine algae[49] were aligned by MUSCLE[36] and by manually on MEGA 7.0.21[50]. The alignment of 87 amino acid positions was subjected to maximum-likelihood analysis and Bayesian interference. For evolutionary model for Bayesian interference and maximum-likelihood analysis, LG + I + G model was selected by ModelTest-NG[43]. The maximum-likelihood analysis was conducted with 1,000 replicates of bootstrap analyses[44] using RAxML-NG[45]. Bayesian interference was performed using MrBayes 3.2.6[46] with 11,000,000 generations of Markov chain Monte Carlo iterations; the first 25% of the generations were discarded as burn-in.

The gene synteny near *RLS1/rlsD* ortholog in volvocine algae were defined based on BLASTP search (E-value < 1e-10) using amino acid sequences of genes near *RLS1/rlsD* ortholog in genome sequences of *V. carteri*, *Y. unicocca*, *G. pectorale* and *C. reinhardtii* and *reg* cluster genes in *V. carteri* and *Y. unicocca*[12,49,51] against gene models of *A. gubernaculifera*. For gene model of *Y. unicocca*, gene models near *RLS1/rlsD* ortholog and *reg* cluster genes predicted in a previous study[49] (accession number: KU257988.1) was used. According to phylogenetic analysis and gene synteny, Agub_g12775 was identified as *RLS1/rlsD* ortholog. BLASTP search (E-value < 1e-10) using genes near Agub_g12775 in *A. gubernaculifera* genome as queries was also conducted against gene models of *V. carteri* v2.1, *C. reinhardtii* v5.6, *G. pectorale* and *Y. unicocca* to confirm the synteny.

Identification and analysis of orthologs of genes involved in embryogenesis in *Volvox carteri*

The amino acid sequences of genes involved in inversion in *V. carteri*, InvA[4] (accession number: BAC77722.1), InvB[6] (accession number: BAH28849.1) and InvC[5] (accession number: BAH03159.1), and genes involved in asymmetric divisions during embryogenesis in *V. carteri*, GlsA[7] (accession number: AAD26632.1) and Hsp70A[52] (accession number: AAZ04921.1), were used as queries in BLASTP search (E-value < 1e-10) against gene models of *A. gubernaculifera*, *Y. unicocca* and *Eudorina* sp. described above as well as *C. reinhardtii*, *T. socialis*, *G. pectorale* and *V. carteri*

(Supplementary Table 4). A single putative ortholog for each gene in each species was found with identity of sequences higher than other hit genes, except for InvC ortholog in *G. pectorale* and GlsA ortholog in *T. socialis*.

As gene models of *InvA* orthologs in *T. socialis*, *G. pectorale*, *A. gubernaculifera* and *Eudorina* sp. were thought to be incomplete, cDNA sequences were sequenced. *A. gubernaculifera* NIES-4017, *Eudorina* sp. strain NIES-4018, *Gonium pectorale* strain NIES-2863 and *Tetrabaena socialis* strain NIES-571 (from Microbial Culture Collection at the National Institute for Environmental Studies, NIES, http://mcc.nies.go.jp/)[26] were cultured in 10 mL of VTAC medium[26,53] in screw-capped tubes (18 × 150 mm) at 25°C on a 12-h light/12-h dark schedule under cool-white fluorescent lamps at an intensity of 50–90 $\mu mol \cdot m^{-2} \cdot s^{-1}$ and used for mRNA isolation. The polyadenylated mRNAs from concentrated cells of each species were isolated using Dynabeads mRNA Purification Kit (Invitrogen, Carlsbad, CA, USA) and then reverse transcribed with Superscript III reverse transcriptase (Invitrogen) according to the manufacture's protocols. For *InvA* ortholog in *G. pectorale* and *Eudorina* sp., The amplification of cDNA sequences was conducted using KOD FX Neo (TOYOBO Co. Ltd., Osaka, Japan), specific primers designed based on genome data (Supplementary Table 5), with following PCR schedule: 94°C for 2 min, following 35 cycles of 98°C for 10 s and 68°C for 40 s. PCR products were purified using illustra GFX PCR DNA and Gel Band Purification Kit (GE healthcare, Little Chalfont, UK) according to the manufacture's protocol. Sequencing of the purified PCR products was carried out using an ABI PRISM 3100 Genetic Analyser (Applied Biosystems, Foster City, CA, USA) with BigDye Terminator v3.1 Cycle Sequencing Kit (Applied Biosystems). For *InvA* orthologs in *T. socialis* and *A. gubernaculifera*, partial cDNA sequences were firstly amplified by nested PCR using TaKaRa Taq (Takara Bio Inc., Shiga, Japan) and degenerate primers (Supplementary Table 5) with following PCR schedule: 35 cycles of 95°C for 2 min, 46°C for 2 min, and 66°C for 3 min, followed by 72°C for 15 min. Purification and sequencing the PCR products were conducted as described above. Then, 5′ and 3′

RACE were conducted for full length cDNA sequences using polyadenylated mRNAs isolated as described above and GeneRacer Kit (Invitrogen) and Superscript III reverse transcriptase (Invitrogen) according to the manufacture's protocols. Nested PCR was conducted using GeneRacer primers in the kit and specific primers (Supplementary Table 5), KOD FX Neo (TOYOBO Co. Ltd.) with 'step-down cycle' on the manufacture's protocol of KOD FX Neo. Purification and sequencing the PCR products were conducted as described above.

The amino acid sequences of InvA, InvB, InvC, GlsA and Hsp70A orthologs were aligned by MUSCLE v3.8.31[36] and alignments were corrected manually. The aligned amino acid sequences were subjected to maximum-likelihood analysis. Evolutionary models for maximum-likelihood analysis were selected using ModelTest-NG[43]. The maximum-likelihood analysis was conducted with 1,000 replicates of bootstrap analyses[44] using RAxML-NG[45]. Orthologs in *C. reinhardtii* were treated as outgroup based on the previous phylogenetic relationships of the volvocine algae[54].

Protein model-based search of ECM genes

The identification of genes in MMP (matrix metalloprotease) family, which is one of expanded ECM gene families in *V. carteri*, was conducted based on methods in a previous study[12]. The MMP domains, coordinate with Pfam metalloprotease domain (PF05548, Peptidase M11), of all MMP genes listed in previous studies[11,12] with available protein model (44 genes in *C. reinhardtii*, 35 genes in *G. pectorale* and 98 genes in *V. carteri*) were aligned by MUSCLE[36] and by manually. The hidden Markov model for MMP domains was built using the aligned sequences by "hmmbuild" in HMMER 3.3 (http://hmmer.org/) and used for protein search against gene models of *C. reinhardtii* v5.6, *T. socialis*, *G. pectorale*, *A. gubernaculifera*, *Y. unicocca*, *Eudorina* sp. and *V. carteri* (v2.1, v2.0 and v1.0) (Supplementary Table 1) by "hmmscan" (E-value < 1e-5) in HMMER. For *V. carteri* v2.0 and v1.0 genome, gene models which are not included in v2.1 genome were counted. Some MMP domains were thought to be separated into two gene models in *Y. unicocca* and *Eudorina* sp. Such

gene models were connected and treated as one gene.

The identification of pherophorin genes, which are also expanded ECM genes in *V. carteri*, were conducted by the essentially the same methods with those for MMP genes. However, typical pherophorin proteins have two pherophorin domains, A-type domain and B-type (pheromone-like) domain[10,55], which are not distinguished in Pfam domain model (PF12499, DUF3707). Therefore, pherophorin domains of pherophorin genes listed in previous studies[11,12] with available protein model (35 genes in *C. reinhardtii*, 20 genes in *G. pectorale* and 75 genes in *V. carteri*, total 225 domains) were first classified into A-type and B-type based on the similarity of amino acid sequences and then used to build the protein models. In addition to A-type and B-type domain models, Pfam domain model (PF12499, DUF3707) were also used for protein search against genome data of volvocine species. As 11 pherophorin genes in *G. pectorale* listed in the previous study[12] were not found in gene models from NCBI or Phytozome, amino acid sequences of pherophorin domains of these genes were deduced manually based on TBLASTN search (E-value < 1e-10) using amino acid sequences of previously reported pherophorin genes.

Cell-type RNA-seq analysis

Separation of somatic and reproductive cells from *A. gubernaculifera* was performed as summarized in Fig. 3a. Mature vegetative colonies before embryogenesis in synchronous culture (ZT 9 in Fig. 1f) were collected by centrifugation at 2,500 rpm for 3 min and homogenized to single cells by strong pipetting. For isolation of somatic cells, the homogenized culture was diluted by 12% Percoll (Sigma-Aldrich, St. Louis, MO, USA) in VTAC medium[26,53], put in 15 mL centrifuge tubes and centrifuged at 3,500 rpm for 10 min. The cells at the upper layer were collected, diluted by 10% Percoll in VTAC medium and centrifuged at 3,500 rpm for 5 min. The sedimented cells after second centrifugation were collected, washed by VTAC medium and used as somatic cell samples. For isolation of reproductive cells, the homogenized culture was diluted by 16% Percoll in VTAC

medium, put in 15 mL centrifuge tubes and centrifuged at 3,500 rpm for 10 min. The sedimented cells were collected, washed by VTAC medium and used as reproductive cell samples. For both somatic and reproductive cells, three samples were prepared from different flasks and immediately used for RNA isolation. A part of cells in each sample were fixed by 0.35% glutaraldehyde, mounted on hemocytometer and observed with a BX53 microscope (Olympus, Tokyo, Japan) to measure the percentage of somatic and reproductive cells of each sample (Supplementary Table 2). In this method, we obtained "somatic cell samples" and "reproductive cell samples", which contained > 70% somatic cells and > 95% reproductive cells, respectively, in total cell number (Fig. 3a, Supplementary Table 2).

The somatic or reproductive cell samples were mixed with RLT buffer in RNeasy Plant Mini Kit (Qiagen, Venlo, Limburg, Netherlands) and ceramic beads in a conical-bottom 2-mL microcentrifuge tube and then vibrated in 25 Hz for 8 min using a Mixer Mill MM300 (Retsch, Haan, Germany). Total RNA in mixture were isolated using RNeasy Plant Mini Kit according to the manufacture's protocol. DNA contamination was removed using TURBO DNA-*free* Kit (Applied Biosystems). Illumina paired-end libraries (library sizes with approx. 500 bp) were constructed using NEBNext Ultra Directional RNA Library Prep Kit for Illumina (New England Biolabs, Ipswich, MA, USA) according to the manufacture's protocol. These libraries were sequenced using Illumina MiSeq sequencer (Illumina). 1.6–2.1 M paired-end reads (250 bp × 2) were generated from each sample (Supplementary Table 2). These reads were processed by Trimmomatic 0.39[56] and PRINSEQ lite 0.20.4[57] for removing adaptor sequences, poly A/T tails, low quality bases and very short reads (less than 30 bases). The processed reads were aligned to *A. gubernaculifera* genome assembly described above using HISAT2 version 2.2.0[58]. Mapped reads were assigned to gene models described above using featureCounts (part of Subread 2.0.0)[59]. 81–82% of high-quality reads were uniquely mapped to 13,198 gene models (Supplementary Table 2) and 515 gene models with no expression in any samples were excluded from further analysis.

Raw read counts were then subjected to differential expression analysis using DESeq2[60]. The genes with differential expression were detected by Wald test between somatic and reproductive cells with a false discovery rate (FDR) < 0.05. Based on this analysis, we identified 2,529 "somatic genes" and 2,328 "reproductive genes", which have significantly higher expression in somatic or reproductive cells (Supplementary Figs. 8, 9, Supplementary Data 3). The threshold-based Wald tests were also conducted with log_2 fold change threshold = 1 ($log_2 2$) or 2.322 ($log_2 5$) in DESeq2 options with a FDR < 0.05. Genes having greater than five-fold significant expression ratio were classified as "somatic-specific" or "reproductive-specific", genes having greater than two-fold significant expression ratio (but less than five-fold) were classified as "somatic-biased" or "reproductive-biased (Supplementary Figs. 8, 9, Supplementary Data 3). The remaining genes without significant difference in expression (FDR > 0.05) were classified as "no difference" (Supplementary Figs. 8, 9, Supplementary Data 3).

The criteria for "specific" and "biased" correspond to those in the previous RNA-seq study on *V. carteri*[24], which showed that nearly half of the expressed genes in *V. carteri* have greater than two-fold expression ratio ("specific" or "biased") and 40% genes have greater than five-fold expression ratio ("specific")[24]. Another RNA-seq study also showed that nearly 55% genes have greater than two-fold expression ratio in *V. carteri*[61]. By contrast, only 893 genes (7%) showed greater than two-fold expression ratio ("specific" or "biased") and 148 genes (1%) showed greater than five-fold expression ratio ("specific") in *A. gubernaculifera*. This situation may have resulted from the difference in isolation of somatic and reproductive cells between *V. carteri* and *A. gubernaculifera*. Though the somatic cells and gonidia are able to be separated completely in *V. carteri*[24,61], somatic and reproductive cells were difficult to separate and both cell samples contained another type cells to some extent in *A. gubernaculifera* (Supplementary Table 2). TPM (transcripts per million)[62] was also calculated to estimate the abundance of transcripts.

Comparison of expression pattern between somatic and reproductive cells

GO (gene ontology) enrichment analysis of cell-type RNA-seq data was conducted using Blast2GO[63]. GO terms were assigned with BLASTP search results (E-value < 1e-3) by DIAMOND v2.0.2.140[64] using all expressed genes in *A. gubernaculifera* as queries against NCBI nonredundant database and InterProScan[65] output with ANNEX[66] and GO-slim[67] options on Blast2GO. Enrichment of GO terms in "somatic genes" and "reproductive genes" were detected by two-sided Fisher's exact test with an odds ratio > 1 and a FDR < 0.05 as criteria.

The *V. carteri* genes involved in motility, photosynthesis, central carbon metabolism, nucleotide-sugar metabolism and glycosyltransferases, whose expressions in somatic cells and reproductive cells (gonidia) were reported in the previous study[24], and their orthologs in *C. reinhardtii* were used as queries in BLASTP search (E-value < 1e-10) against gene models of *A. gubernaculifera* described above. Some genes which were not included in the previous datasets[24] were added to queries based on previous studies[68–70]. The LHCII and LHCI genes were identified based on phylogenetic analysis of LHC genes in *A. gubernaculifera*, *C. reinhardtii* and *V. carteri* (Supplementary Fig. 15). The expression in somatic and reproductive cells and classification of hit putative orthologs were listed in Supplementary Data 4–7. Metabolic pathways were deduced based on previous studies[24,70,71]. The expression levels of genes involved in multicellular traits, VARL genes, orthologous genes involved in embryogenesis in *V. carteri* (InvA, InvB, InvC, GlsA and Hsp70A), and ECM genes (MMPs and pherophorins), were also listed in Supplementary Data 8.

Phylogenetic analysis of somatic-specific transcription factors in *Astrephomene gubernaculifera*

The amino acid sequences of three somatic-specific genes encoding transcription factors, Agub_g2945, Agub_g5284 and Agub_g8265, were used as queries for BLASTP search (E-value < 1e-10) against genome data of 15 algal/plant species (Supplementary Table 4). Based on the BLASTP results and phylogeny of MYB transcription factors and RWP-RK transcription factors in

previous studies[72–74], 75, 43 and 127 closely related genes in these species were selected for the phylogenetic analyses of Agub_g2945, Agub_g5284 and Agub_g8265, respectively. The DNA binding domain of these genes were aligned by MUSCLE[36]. The alignments of 105, 87, 49 amino acid positions, respectively, were subjected to maximum-likelihood analysis. For evolutionary model for maximum-likelihood analysis, LG + I + G model or LG + I + G +F model were selected for the datasets of MYB transcription factors including Agub_g2945 or Agub_g5284, respectively, and LG + G model was selected for the dataset of RWP-RK transcription factors by ModelTest-NG[43]. The maximum-likelihood analysis was conducted with 1,000 replicates of bootstrap analyses[44] using RAxML-NG[45].

Supplementary Table 1. Summary statics for genomes of volvocine green algae used in this study.

Species	*Chlamydomonas reinhardtii* v5.5	*Tetrabaena socialis*	*Gonium pectorale*	*Astrephomene gubernaculifera*	*Yamagishiella unicocca* (plus)	*Eudorina* sp. (female)	*Volvox carteri* v2.1
Genome size (Mb)	111.1	135.8	148.8	103.8	134.2	184.0	131.2
Scaffold N50 (Mb)	7.78	0.14	1.27	1.50	0.66	0.56	2.6
Number of contigs/scaffolds	54	5,858	2,373	206	1,461	3,180	434
% G and C	64.1	66.0	64.5	59.9	61.0	61.0	56.1
Protein coding loci	17,741	15,513	17,984	13,713	21,231[a]	28,661[a]	14,247
References	75	13	12	this study	23	23	11

[a] Predicted in this study, by AUGUSTUS v3.3.3[76] using the default parameter of *V. carteri*.

Supplementary Table 2. Characteristics of the samples for cell-type RNA-seq in *Astrephomene gubernaculifera* and their reads mapping statistics.

Sample	somatic1	somatic2	somatic3	reproductive1	reproductive2	reproductive3
Percentage of somatic cells	74.0%	70.7%	70.7%	3.9%	3.5%	2.9%
Percentage of reproductive cells	25.5%	28.7%	28.9%	96.1%	96.5%	97.1%
Number of total reads	1,800,628	1,932,566	2,081,114	2,179,646	1,663,646	1,765,606
Number of high-quality reads	1,766,185 (98.09%)	1,892,324 (97.92%)	2,032,021 (97.64%)	2,135,021 (97.95%)	1,629,527 (97.95%)	1,725,977 (97.76%)
Number of reads uniquely mapped to genome	1,552,330 (86.21%)	1,642,394 (84.99%)	1,768,757 (84.99%)	1,891,595 (86.78%)	1,435,092 (86.26%)	1,522,967 (86.26%)
Number of reads uniquely mapped to gene models	1,491,965 (82.86%)	1,579,356 (81.72%)	1,700,723 (81.72%)	1,801,205 (82.64%)	1,364,596 (82.02%)	1,454,112 (82.36%)

Supplementary Table 3. GO terms enriched in somatic and reproductive genes in *Astrephomene gubernaculifera*.

GO ID	GO Name	GO Category	FDR
Enriched terms in somatic genes			
GO:0007165	signal transduction	BIOLOGICAL_PROCESS	7.38E-11
GO:0007154	cell communication	BIOLOGICAL_PROCESS	1.21E-10
GO:0050794	regulation of cellular process	BIOLOGICAL_PROCESS	3.50E-10
GO:0016020	Membrane	CELLULAR_COMPONENT	3.50E-10
GO:0051716	cellular response to stimulus	BIOLOGICAL_PROCESS	5.44E-10
GO:0016021	integral component of membrane	CELLULAR_COMPONENT	2.86E-09
GO:0050789	regulation of biological process	BIOLOGICAL_PROCESS	6.46E-09
GO:0005794	Golgi apparatus	CELLULAR_COMPONENT	5.96E-08
GO:0016301	kinase activity	MOLECULAR_FUNCTION	1.00E-07
GO:0065007	biological regulation	BIOLOGICAL_PROCESS	5.10E-07
GO:0005856	Cytoskeleton	CELLULAR_COMPONENT	1.86E-05
GO:0030705	cytoskeleton-dependent intracellular transport	BIOLOGICAL_PROCESS	1.55E-04
GO:0003700	DNA-binding transcription factor activity	MOLECULAR_FUNCTION	2.67E-04
GO:0070085	Glycosylation	BIOLOGICAL_PROCESS	3.80E-04
GO:0140110	transcription regulator activity	MOLECULAR_FUNCTION	5.41E-04
GO:0016772	transferase activity, transferring phosphorus-containing groups	MOLECULAR_FUNCTION	6.15E-04
GO:0036211	protein modification process	BIOLOGICAL_PROCESS	6.92E-04
GO:0043412	macromolecule modification	BIOLOGICAL_PROCESS	1.04E-03
GO:0012505	endomembrane system	CELLULAR_COMPONENT	3.14E-03
GO:0031410	cytoplasmic vesicle	CELLULAR_COMPONENT	3.37E-03
GO:0031982	Vesicle	CELLULAR_COMPONENT	4.35E-03
GO:0006508	Proteolysis	BIOLOGICAL_PROCESS	5.81E-03
GO:0005576	extracellular region	CELLULAR_COMPONENT	6.33E-03
GO:0006486	protein glycosylation	BIOLOGICAL_PROCESS	6.82E-03
GO:0008234	cysteine-type peptidase activity	MOLECULAR_FUNCTION	6.82E-03
GO:0005929	Cilium	CELLULAR_COMPONENT	1.52E-02
GO:0016192	vesicle-mediated transport	BIOLOGICAL_PROCESS	1.75E-02
GO:0071944	cell periphery	CELLULAR_COMPONENT	2.00E-02
GO:0005886	plasma membrane	CELLULAR_COMPONENT	4.67E-02
GO:0055086	nucleobase-containing small molecule metabolic process	BIOLOGICAL_PROCESS	4.94E-02

Supplementary Table 3. Continued.

Enriched terms in reproductive genes			
GO:0006091	generation of precursor metabolites and energy	BIOLOGICAL_PROCESS	8.40E-37
GO:0044281	small molecule metabolic process	BIOLOGICAL_PROCESS	1.85E-29
GO:0042254	ribosome biogenesis	BIOLOGICAL_PROCESS	6.57E-23
GO:0009058	biosynthetic process	BIOLOGICAL_PROCESS	6.57E-23
GO:0015979	Photosynthesis	BIOLOGICAL_PROCESS	1.11E-22
GO:0022613	ribonucleoprotein complex biogenesis	BIOLOGICAL_PROCESS	8.42E-22
GO:0016491	oxidoreductase activity	MOLECULAR_FUNCTION	8.33E-21
GO:0006520	cellular amino acid metabolic process	BIOLOGICAL_PROCESS	1.05E-18
GO:0006082	organic acid metabolic process	BIOLOGICAL_PROCESS	5.17E-18
GO:0019752	carboxylic acid metabolic process	BIOLOGICAL_PROCESS	6.77E-18
GO:0008152	metabolic process	BIOLOGICAL_PROCESS	6.82E-18
GO:0044237	cellular metabolic process	BIOLOGICAL_PROCESS	2.71E-16
GO:0005622	intracellular anatomical structure	CELLULAR_COMPONENT	6.46E-16
GO:0043603	cellular amide metabolic process	BIOLOGICAL_PROCESS	1.02E-13
GO:0006412	Translation	BIOLOGICAL_PROCESS	1.02E-13
GO:0009987	cellular process	BIOLOGICAL_PROCESS	1.02E-13
GO:0043043	peptide biosynthetic process	BIOLOGICAL_PROCESS	1.80E-13
GO:0006518	peptide metabolic process	BIOLOGICAL_PROCESS	2.05E-13
GO:0044085	cellular component biogenesis	BIOLOGICAL_PROCESS	6.23E-13
GO:0043228	non-membrane-bounded organelle	CELLULAR_COMPONENT	8.85E-13
GO:0005730	Nucleolus	CELLULAR_COMPONENT	2.60E-12
GO:0005840	Ribosome	CELLULAR_COMPONENT	1.38E-11
GO:0043229	intracellular organelle	CELLULAR_COMPONENT	1.17E-10
GO:1901566	organonitrogen compound biosynthetic process	BIOLOGICAL_PROCESS	1.57E-10
GO:0034641	cellular nitrogen compound metabolic process	BIOLOGICAL_PROCESS	2.34E-10
GO:0016874	ligase activity	MOLECULAR_FUNCTION	1.80E-09
GO:0071840	cellular component organization or biogenesis	BIOLOGICAL_PROCESS	4.56E-09
GO:0032991	protein-containing complex	CELLULAR_COMPONENT	6.06E-09
GO:0003735	structural constituent of ribosome	MOLECULAR_FUNCTION	6.19E-09
GO:0005739	Mitochondrion	CELLULAR_COMPONENT	1.00E-08
GO:0043226	Organelle	CELLULAR_COMPONENT	1.56E-08
GO:0006457	protein folding	BIOLOGICAL_PROCESS	2.81E-08
GO:0006790	sulfur compound metabolic process	BIOLOGICAL_PROCESS	3.47E-08
GO:0003824	catalytic activity	MOLECULAR_FUNCTION	9.57E-08
GO:0009579	Thylakoid	CELLULAR_COMPONENT	5.88E-07

Supplementary Table 3. Continued.

GO:0005198	structural molecule activity	MOLECULAR_FUNCTION	8.21E-07
GO:0043231	intracellular membrane-bounded organelle	CELLULAR_COMPONENT	3.50E-06
GO:0051082	unfolded protein binding	MOLECULAR_FUNCTION	3.56E-06
GO:0034645	cellular macromolecule biosynthetic process	BIOLOGICAL_PROCESS	1.51E-05
GO:0005488	Binding	MOLECULAR_FUNCTION	2.36E-05
GO:0043227	membrane-bounded organelle	CELLULAR_COMPONENT	4.67E-05
GO:0010467	gene expression	BIOLOGICAL_PROCESS	6.03E-05
GO:0003723	RNA binding	MOLECULAR_FUNCTION	1.08E-04
GO:0006807	nitrogen compound metabolic process	BIOLOGICAL_PROCESS	1.11E-04
GO:0009059	macromolecule biosynthetic process	BIOLOGICAL_PROCESS	1.47E-04
GO:0005634	Nucleus	CELLULAR_COMPONENT	1.47E-04
GO:0043233	organelle lumen	CELLULAR_COMPONENT	1.96E-04
GO:0110165	cellular anatomical entity	CELLULAR_COMPONENT	2.45E-04
GO:0044271	cellular nitrogen compound biosynthetic process	BIOLOGICAL_PROCESS	3.22E-04
GO:0031981	nuclear lumen	CELLULAR_COMPONENT	3.40E-04
GO:0006399	tRNA metabolic process	BIOLOGICAL_PROCESS	6.65E-04
GO:0003676	nucleic acid binding	MOLECULAR_FUNCTION	1.30E-03
GO:0034660	ncRNA metabolic process	BIOLOGICAL_PROCESS	1.65E-03
GO:0005737	Cytoplasm	CELLULAR_COMPONENT	1.86E-03
GO:0007005	mitochondrion organization	BIOLOGICAL_PROCESS	1.92E-03
GO:0043167	ion binding	MOLECULAR_FUNCTION	5.05E-03
GO:0044238	primary metabolic process	BIOLOGICAL_PROCESS	5.10E-03
GO:0044249	cellular biosynthetic process	BIOLOGICAL_PROCESS	6.24E-03
GO:0019843	rRNA binding	MOLECULAR_FUNCTION	6.97E-03
GO:1901576	organic substance biosynthetic process	BIOLOGICAL_PROCESS	7.83E-03
GO:0071704	organic substance metabolic process	BIOLOGICAL_PROCESS	1.08E-02
GO:0016829	lyase activity	MOLECULAR_FUNCTION	1.09E-02
GO:0005829	Cytosol	CELLULAR_COMPONENT	1.23E-02
GO:0090079	translation regulator activity, nucleic acid binding	MOLECULAR_FUNCTION	1.92E-02
GO:0016853	isomerase activity	MOLECULAR_FUNCTION	2.03E-02
GO:0097159	organic cyclic compound binding	MOLECULAR_FUNCTION	2.03E-02
GO:0045182	translation regulator activity	MOLECULAR_FUNCTION	2.07E-02
GO:0051169	nuclear transport	BIOLOGICAL_PROCESS	2.49E-02
GO:1901564	organonitrogen compound metabolic process	BIOLOGICAL_PROCESS	2.65E-02
GO:0005975	carbohydrate metabolic process	BIOLOGICAL_PROCESS	4.33E-02

Red background color: common GO terms between *A. gubernaculifera* and *V. carteri*[24].

Supplementary Table 4. List of genome data used in this study.

Species, version	Reference	Source
Astrephomene gubernaculifera	this study	NCBI (BioProject accession number: PRJDB10253)
Chlamydomonas reinhardtii v5.6	75	Phytozome v12.1 (https://phytozome.jgi.doe.gov/pz/portal.html#!info?alias=Org_Creinhardtii)
Volvox carteri v1.0, v2.0, v2.1	11	Phytozome v12.1 (https://phytozome.jgi.doe.gov/pz/portal.html#!info?alias=Org_Vcarteri)
Gonium pectorale	12	NCBI (https://www.ncbi.nlm.nih.gov/genome/16856)
Tetrabaena socialis	13	NCBI (https://www.ncbi.nlm.nih.gov/genome/66769)
Yamagishiella unicocca	23	NCBI (https://www.ncbi.nlm.nih.gov/genome/53185)
Eudorina sp.	23	NCBI (https://www.ncbi.nlm.nih.gov/genome/69315)
Volvox reticuliferus	77	NCBI (https://www.ncbi.nlm.nih.gov/genome/105015)
Volvox africanus	77	NCBI (https://www.ncbi.nlm.nih.gov/genome/80690)
Chlamydomonas schloesseri	78	NCBI (https://www.ncbi.nlm.nih.gov/genome/98313)
Edaphochlamys debaryana	78	NCBI (https://www.ncbi.nlm.nih.gov/genome/45181)
Arabidopsis thaliana TAIR10	79	Phytozome v12.1 (https://phytozome.jgi.doe.gov/pz/portal.html#!info?alias=Org_Athaliana)
Amborella trichopoda	80	Phytozome v12.1 (https://phytozome.jgi.doe.gov/pz/portal.html#!info?alias=Org_Atrichopoda)

Supplementary Table 4. Continued.

Picea abies	81	ConGenIE (ftp://plantgenie.org/Data/ConGenIE/Picea_abies/v1.0)
Selaginella moellendorffii	82	Phytozome v12.1 (https://phytozome.jgi.doe.gov/pz/portal.html#!info?alias=Org_Smoellendorffii)
Physcomitrella patens v3.0	83	Phytozome v12.1 (https://phytozome.jgi.doe.gov/pz/portal.html#!info?alias=Org_Ppatens)
Marchantia polymorpha v3.1	74	Phytozome v12.1 (https://phytozome.jgi.doe.gov/pz/portal.html#!info?alias=Org_Mpolymorpha)
Klebsormidium nitens	84	NCBI (https://www.ncbi.nlm.nih.gov/genome/54879)
Ostreococcus tauri	85	https://bioinformatics.psb.ugent.be/gdb/ostreococcus/
Cyanidioschyzon merolae	86	http://czon.jp/

Supplementary Table 5. List of specific primers used for amplification and sequencing of *InvA* orthologs.

Designation	Positions[a]	Sequence (5′–3′)
Gonium pectorale		
GpInvA_F1	(5′ UTR)	CCTCTGACTTCTTTCTTGCCGATC
GpInvA_F2	396–418	ACGTGTTATTCGCCACATCTTCG
GpInvA_F3	843–865	CGAGTTCACCAGCAAGCTCCACT
GpInvA_F4	1412–1435	AGCAGATGAAAGCAAAGGGATTCC
GpInvA_F5	1873–1896	GGAGATGTCGGCACTGAGGACTAC
GpInvA_F6	2410–2432	GACAAGATGACGTACCTGCTCGC
GpInvA_R1	692–668[b]	GTAAAGAAGGACAGAAGCTCCTCCG
GpInvA_R2	1182–1158[b]	GTTGGCATACTTCAGGGTGTTGTTG
GpInvA_R3	1589–1568[b]	CTTTCCACAGAGTTCGCCACCT
GpInvA_R4	2352–2331[b]	CTTTGTGCAGGCCTTGAAAACG
GpInvA_R5	2756–2733[b]	CAAGTGATTGTGTTGGCCTTTTCG
GpInvA_R6	(3′ UTR)[b]	AAGTTCGAGCCAGCCATGAAAAG
Eudorina sp.		
EuInvA_F1	(5′ UTR)	TTAAGCTGATCCCTTTGCCTGTTG
EuInvA_F2	390–412	ACGGGTTATTCGGCACATCTTTG
EuInvA_F3	917–939	TCAAGGAGGCCACGCACATTAAC
EuInvA_F4	1359–1381	CAAGGAGAAGGAGGCCAAACGAG
EuInvA_F5	1975–1999	GGCGACTCAATGTTTACGGAAGAAG
EuInvA_F6	2582–2604	CCTCGTGCATCTTCGACTTTGAC
EuInvA_R1	501–478[b]	CAAGTCCTCGTTGTACAGCTCCAG
EuInvA_R2	1174–1150[b]	CGTACTTCAGGGTGTTGTTCGTCTC
EuInvA_R3	1686–1664[b]	GGTGTTCGGCAGGACTTTCGTAC
EuInvA_R4	2222–2200[b]	CTATCGACCGGCTCCACTGTTTC
EuInvA_R5	2646–2622[b]	AATTCGCTCGTCCACACCGTAGTAC
EuInvA_R6	(3′ UTR)[b]	TGATACCAAGCGCACACTTCAAG

Supplementary Table 5. Continued.

degenerated primers		
InvA-F11	1141–1163	GAYTTYGARGARACIAAYAAYAC
InvA-F12	1183–1205	GCITGYAARATHAARAAYCARCC
InvA-F13	1405–1427	GARGARCARATGAARGCIAARGG
InvA-F0	2311–2330	GCIGTITTYAARGCITGYAC
InvA-F1	2332–2351	AARGARGAYTTYAARGAYGT
InvA-F2	2521–2540	ATGGCIACITTYGGIACIAA
InvA-R1	2801–2782[b]	CCRTTYTCDATIGCRTGRTT
InvA-R2	2867–2848[b]	TTIACICCIGCYTGCATCCA
InvA-R3	2972–2953[b]	ACYTCIGCIACCATRAACCA
Astrephomene gubernaculifera		
InvA Primer SR1	2383–2359[b]	GTAGGTAGGTCATCTTGTCTGAGGC
InvA Primer SR2	2556–2532[b]	CTCCTCTGGGTCCTCAAAGTCGAAG
InvA Primer SR3	2587–2563[b]	CAATCCGCTCGTCCACACCGTAATA
AgInvA_R1	1522–1498[b]	CAATGACCTGATCCGTCGGGATGCT
AgInvA_R2	1554–1532[b]	CCGCGTACCGAACCCCTTCAACA
AgInvA_R3	709–685[b]	TGGATCGGTTGTCCATGCACAGGTT
AgInvA_R4	782–758[b]	ACGGTGCGCTGGATGGTGATAGTGA
AgInvA_F1	2564–2588	ATTACGGTGTGGACGAGCGGATTGA
AgInvA_F2	2675–2698	AGGCGTCGAAGGACAACACGATCA
AgInvA_F5	(3′ UTR)	GGGTACACTCTGTTTCTGCACGCACA
AgInvA_F6	(3′ UTR)	CCACAAGGTCTCCGCTAGTATGATAG
Tetrabaena socialis		
TsInvA_F1	95–119	TGAACTTTGATGACGAAACCAGACA
TsInvA_R1	405–384[b]	CGCGAAAATGTGCTGGATGACC
TsInvA_F2	259–282	GAGGCGGTTTTCAAGGGTTACAAC
TsInvA_R2	2462–2439[b]	AGCAGGTAGGTCATCTTGTCGGAG
TsInvA_R3	164–141[b]	CGTAGCTGGAGAAGGGTGTTCTTG

Supplementary Table 5. Continued.

TsInvA_R4	287–263[b]	GTTGCGTTGTAACCCTTGAAAACCG
TsInvA_F3	2764–2786	TCCAAGGACAGCACCATCACCTG
TsInvA_F4	2806–2829	AACATGTCCATCGGCCTGTTGTAC
TsInvA_R5	2599–2578[b]	CGATCATGTTGGTGCCGAACGT
TsInvA_F5	(3′ UTR)	CCGGGTCTGCAATGGTTCGTGG
TsInvA_F6	(5′ UTR)	GCCTGGGAAAGAAACTGCATCATC
TsInvA_R6	(3′ UTR)[b]	ACGAACCATTGCAGACCCGGTG
TsInvA_F7	909–930	CAAGGAGGCGACCCACATCAAC
TsInvA_F8	1482–1502	CTCCATCCCCAGCGACCAGGT
TsInvA_R7	1838–1817[b]	CCCACAAAGAAGTCGTCCGTCC

[a] Coordinate number from CDS of cDNA sequence determined in this study.

[b] Reverse primer.

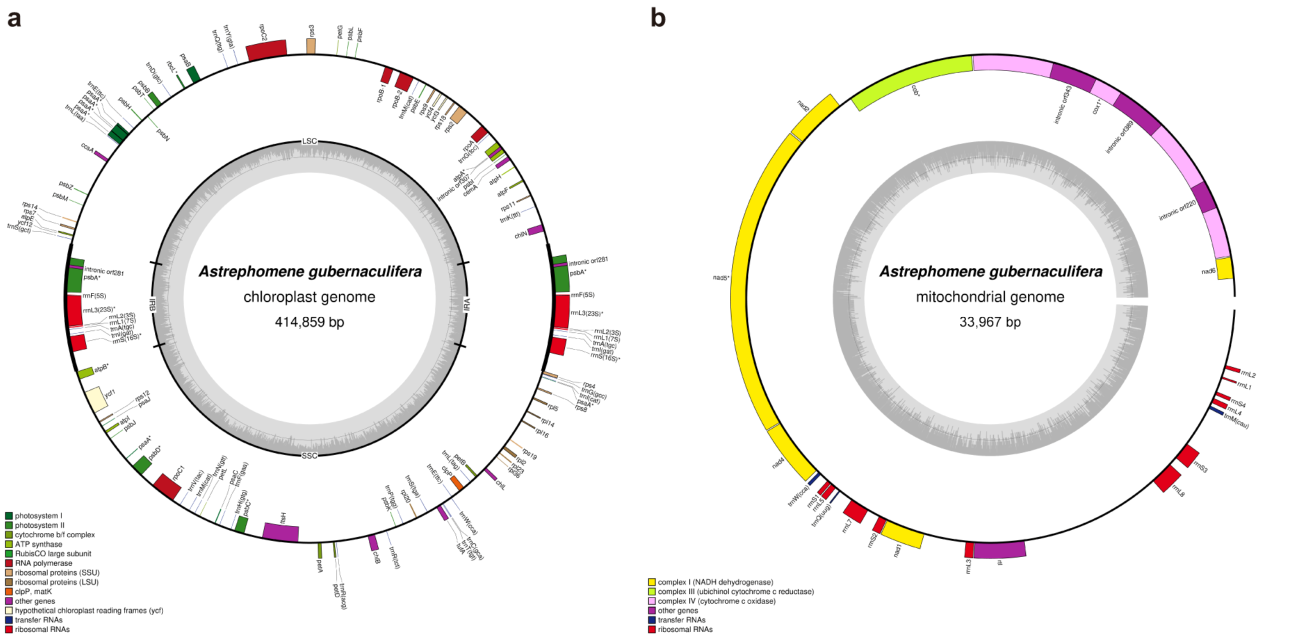

Supplementary Figure 1. Genetic maps of the *Astrephomene gubernaculifera* organelle genome. (**a**) Plastid genome. (**b**) Mitochondrial genome. The maps were generated by ORDRAW[87] on CHLOROBOX (https://chlorobox.mpimp-golm.mpg.de/index.html). The *A. gubernaculifera* plastid genome is a circular DNA molecule, while it is not determined whether the mitochondrial genome is circular or linear in this study. Asterisk indicates the presence of introns in each gene. Transfer RNA (tRNA) genes are designated by the single-letter abbreviation of the amino acid they specify, and three letters in parentheses of each gene represent the anticodons.

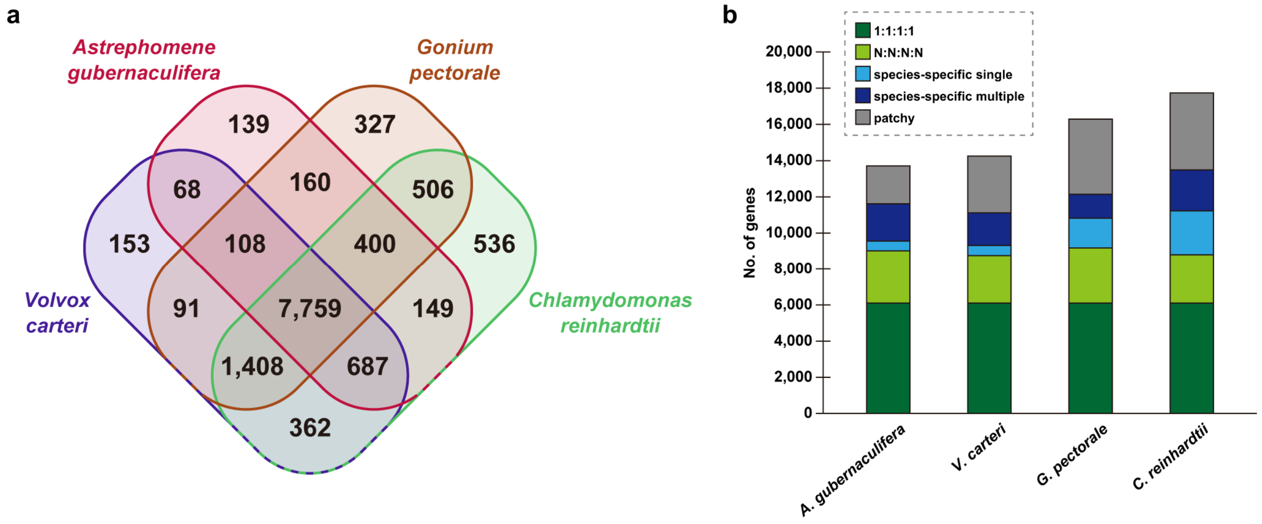

Supplementary Figure 2. The comparison of the orthogroup contents within the volvocine algae. (**a**) Venn diagram showing the number of orthogroups (by OrthoFinder[35]) shared by *A. gubernaculifera*, *V. carteri*, *G. pectorale* and *C. reinhardtii* genomes. (**b**) Gene contents in terms of orthogroups of *A. gubernaculifera*, *V. carteri*, *G. pectorale* and *C. reinhardtii* genomes. “1:1:1:1” indicates orthologs shared by four species as single copies, “N:N:N:N” indicates orthologs shared by four species as multiple copies, “patchy” indicates an ortholog shared by only two or three species.

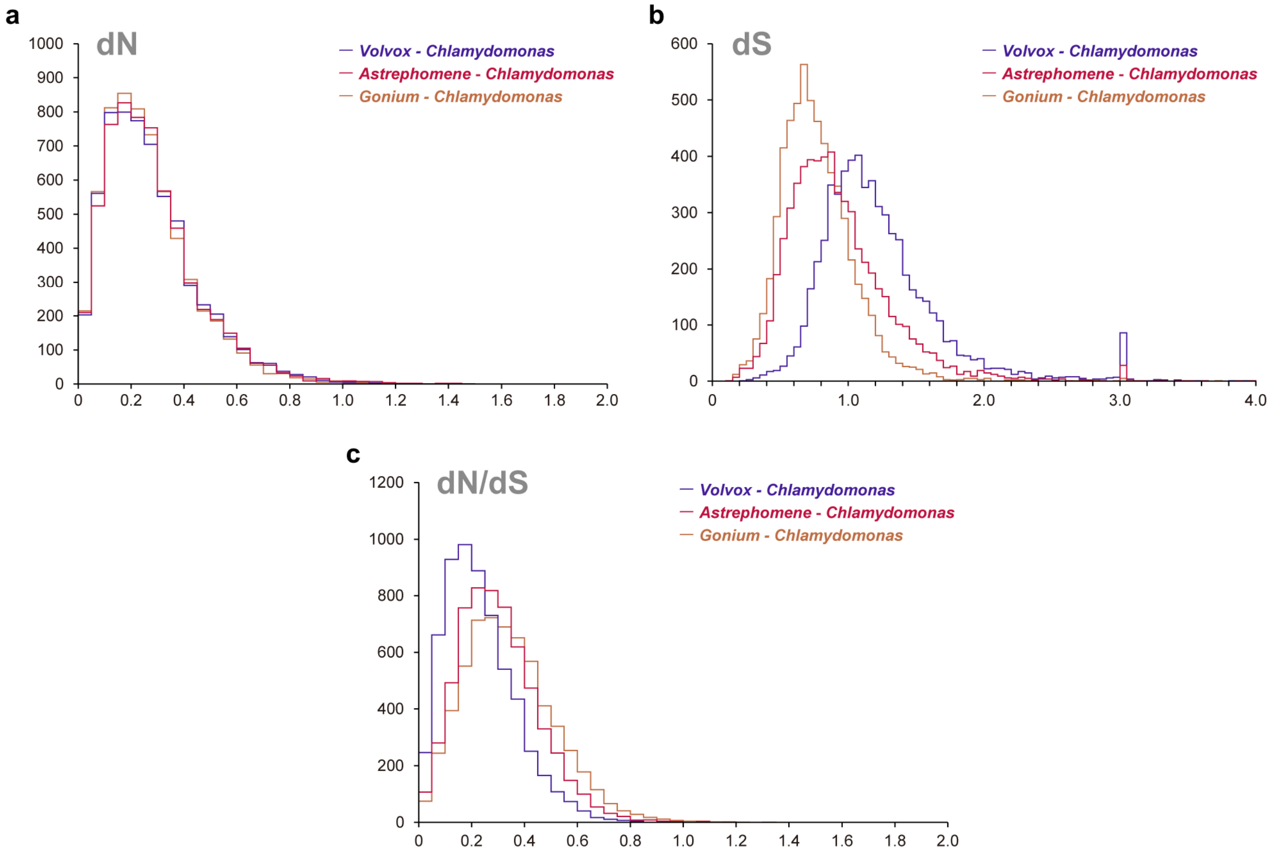

Supplementary Figure 3. Comparison of the genome wide distributions of substitution rate. Histograms showing the distributions of (**a**) dN, (**b**) dS and (**c**) dN/dS values for 6,091 1:1:1:1 orthologs (Supplementary Fig. 2b) from pairwise comparison between *V. carteri* and *C. reinhardtii* (blue), *A. gubernaculifera* and *C. reinhardtii* (red) and *G. pectorale* and *C. reinhardtii* (yellow), respectively.

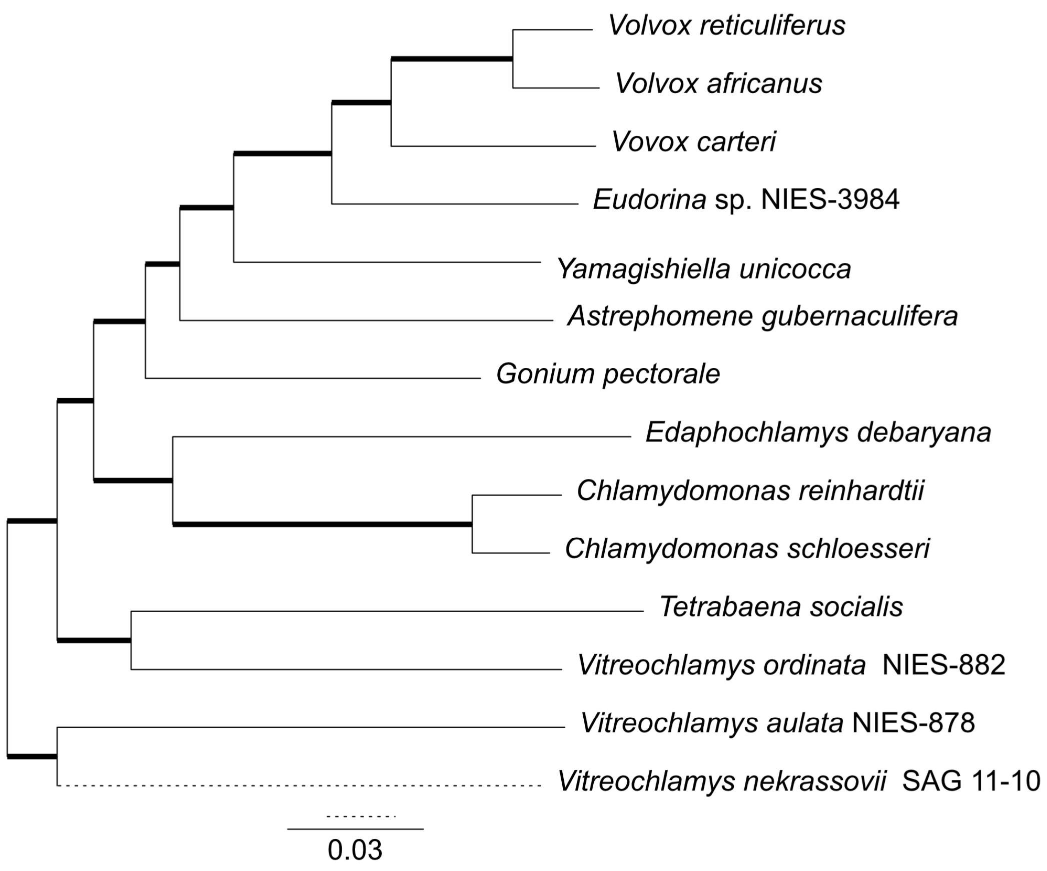

Supplementary Figure 4. Maximum-likelihood phylogenetic tree based on 371 nuclear genome-encoded proteins. The dataset was composed of 140,917 amino acids of 14 volvocine species. All nodes were supported with bootstrap values = 100 and Bayesian posterior probabilities = 1.00 (bold line).

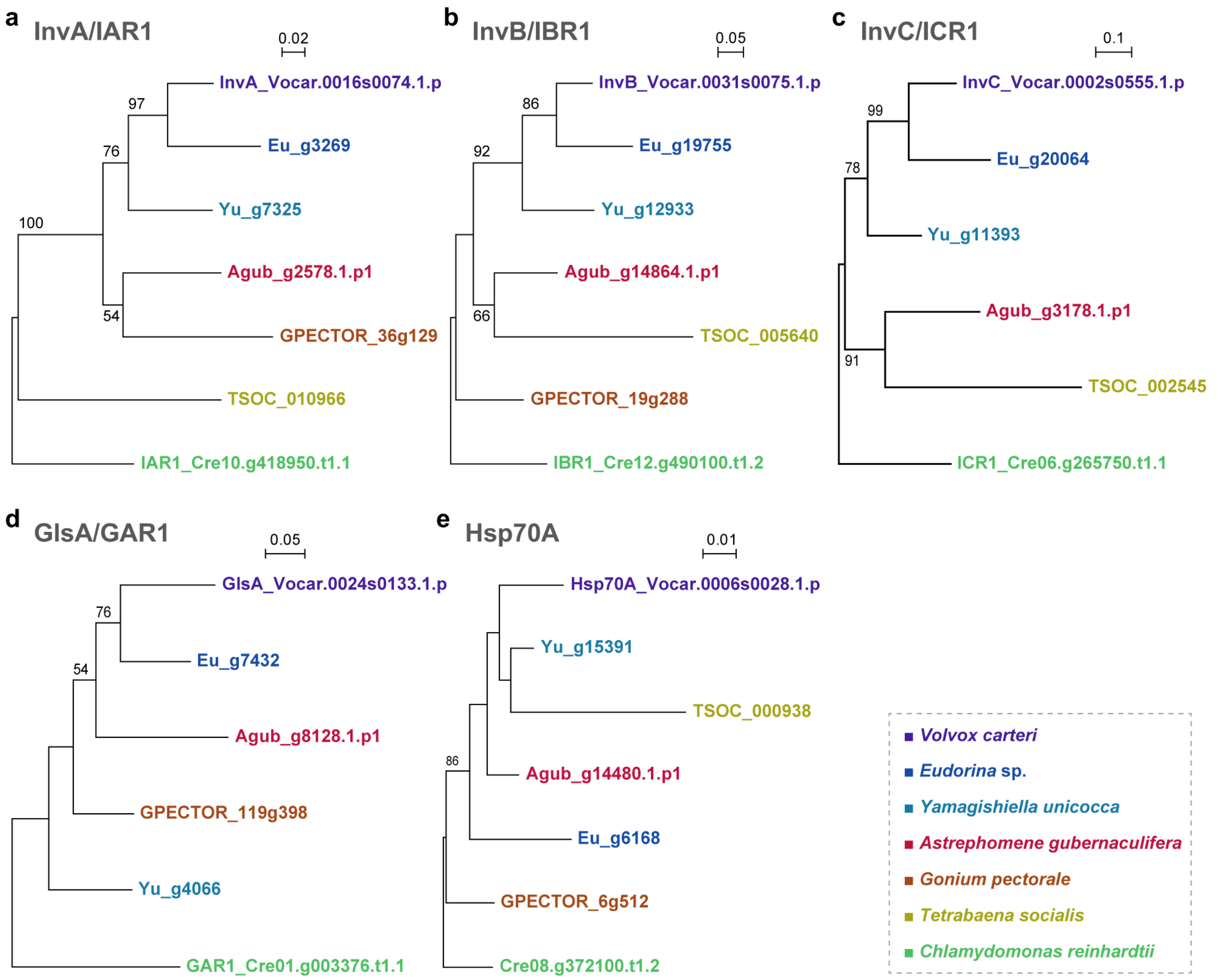

Supplementary Figure 5. Maximum-likelihood phylogenetic trees of orthologs of five genes involved in embryogenesis in *Volvox carteri*. Six or seven volvocine orthologs were analyzed in each tree. The bootstrap values (≥ 50%) are shown at branches. The colors indicate species according to the color key at the lower right. (**a**) InvA/IAR1 orthologs (1,007 amino acid positions, DAYHOFF + I + G + F model). (**b**) InvB/IBR1 orthologs (347 amino acid positions, JTT + I + G + F model). (**c**) InvC/ICR1 orthologs (391 amino acid positions, LG + G + F model). (**d**) GlsA/GAR1 orthologs (634 amino acid positions, DAYHOFF + G + F model). (**e**) Hsp70A orthologs (653 amino acid positions, LG + I + G + F model).

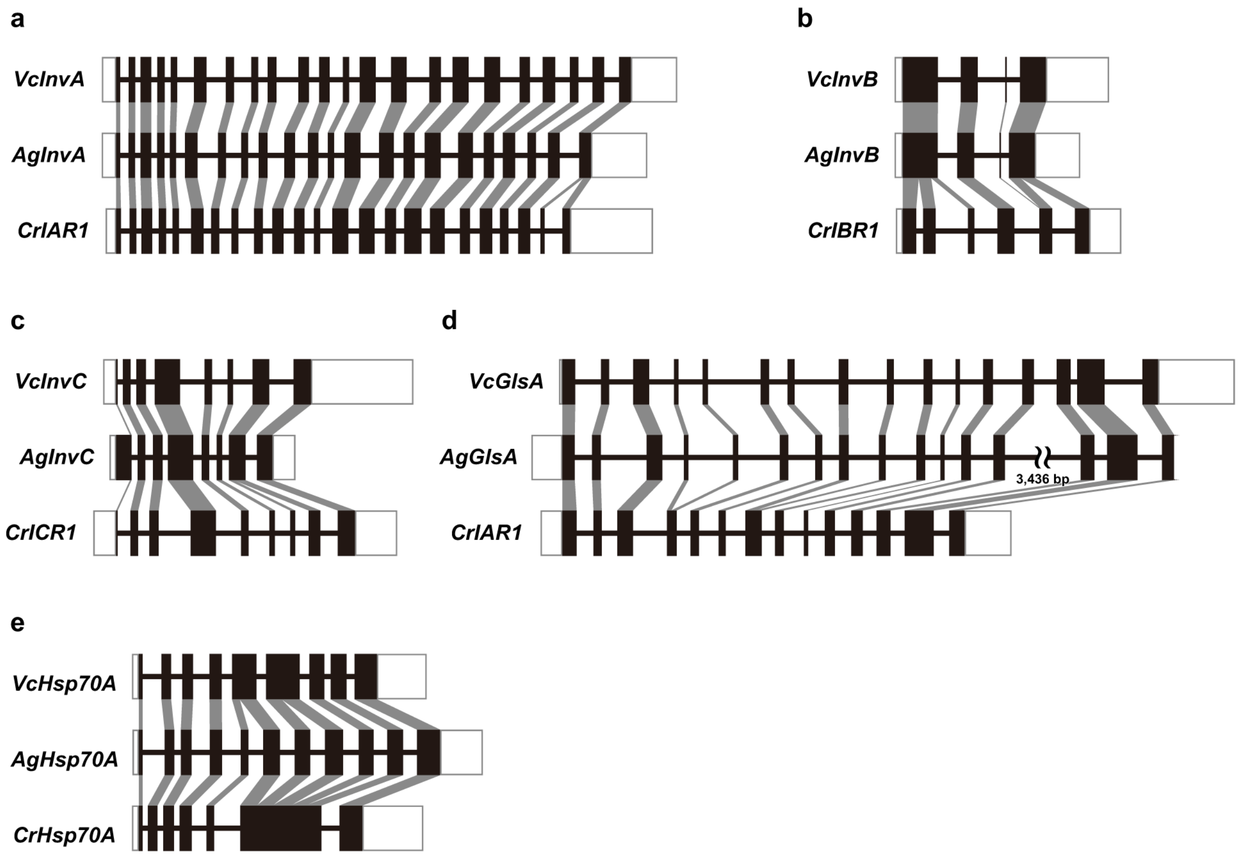

Supplementary Figure 6. Comparison of the exon–intron structure of orthologs of five genes involved in embryogenesis in *Volvox carteri*. The exon–intron structures from gene model in *V. carteri* (*Vc*), *Astrephomene gubernaculifera* (*Ag*) and *Chlamydomonas reinhardtii* (*Cr*) are shown in line. The thick and thin lines correspond to exons and introns respectively, black thick lines indicate CDS and white thick line indicate 5′ and 3′ UTRs. (**a**) *InvA*/*IAR1* orthologs. (**b**) *InvB*/*IBR1* orthologs. (**c**) *InvC*/*ICR1* orthologs. (**d**) *GlsA*/*GAR1* orthologs. (**e**) *Hsp70A* orthologs.

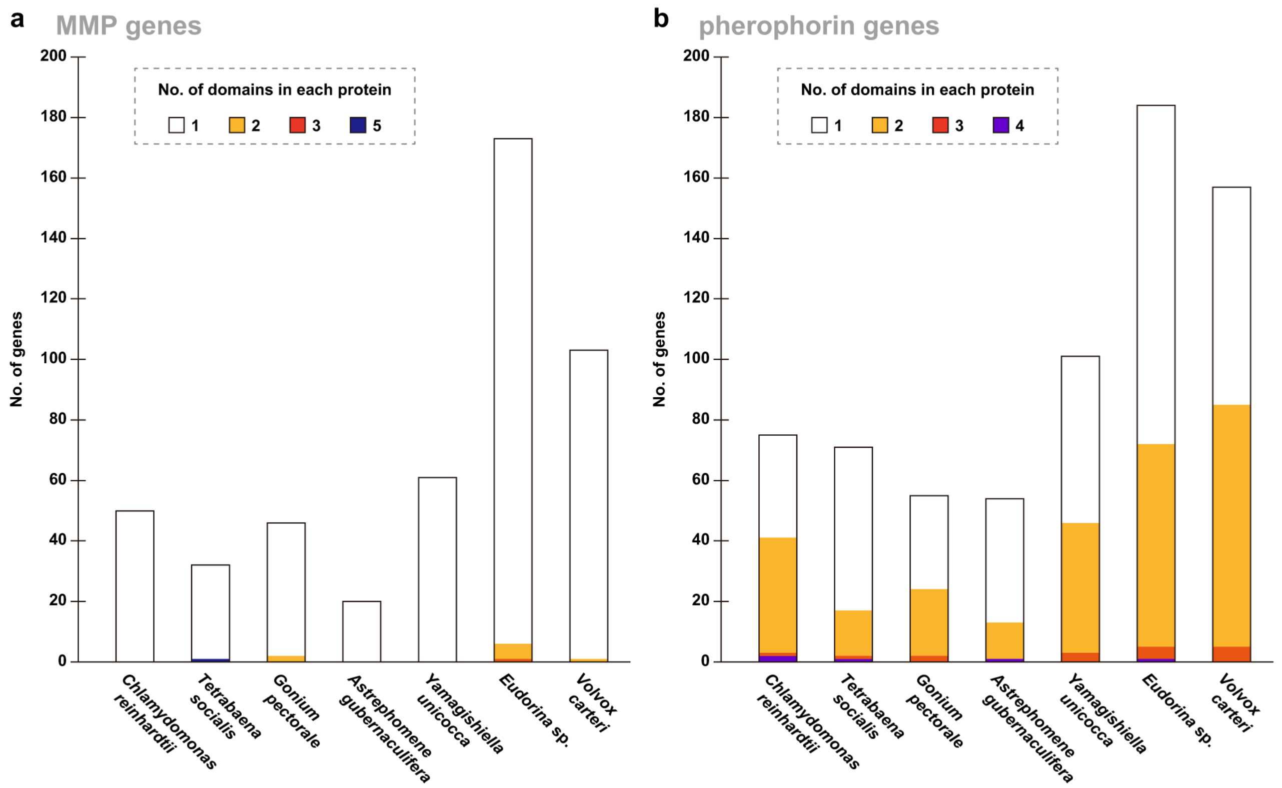

Supplementary Figure 7. The number of ECM genes in volvocine green algae. (**a**) Stacked bar graph of the number of MMP genes (Supplementary Data 1). (**b**) Stacked bar graph of the number of pherophorin genes (Supplementary Data 2). The colors indicate the number of MMP or pherophorin domains in each transcript, according to the color key at the upper left.

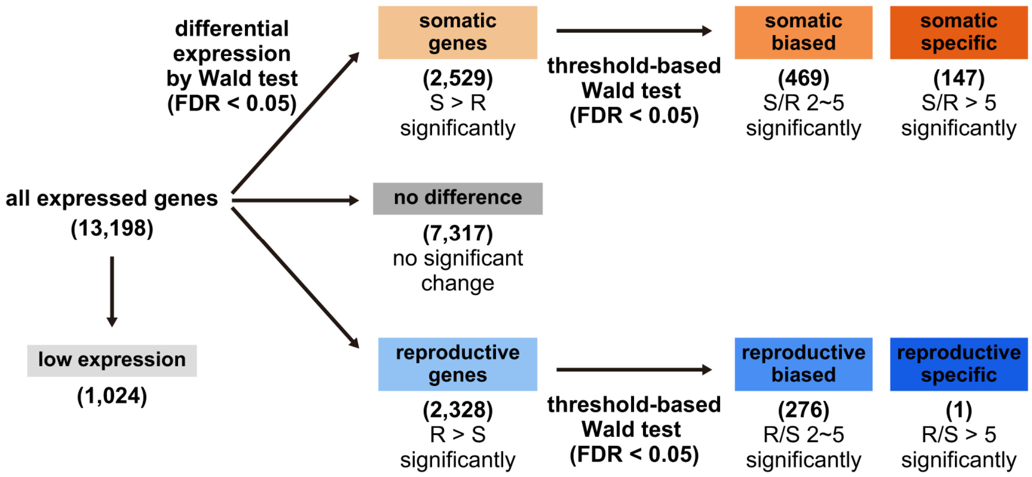

Supplementary Figure 8. Classification of genes by cell-type expression in *Astrephomene gubernaculifera*. All expressed genes are first classified by differential expression analysis using DESeq2, and then the differential expressed genes are further classified by fold changes (see Supplementary Methods for detail). R: reproductive expression, S: somatic expression.

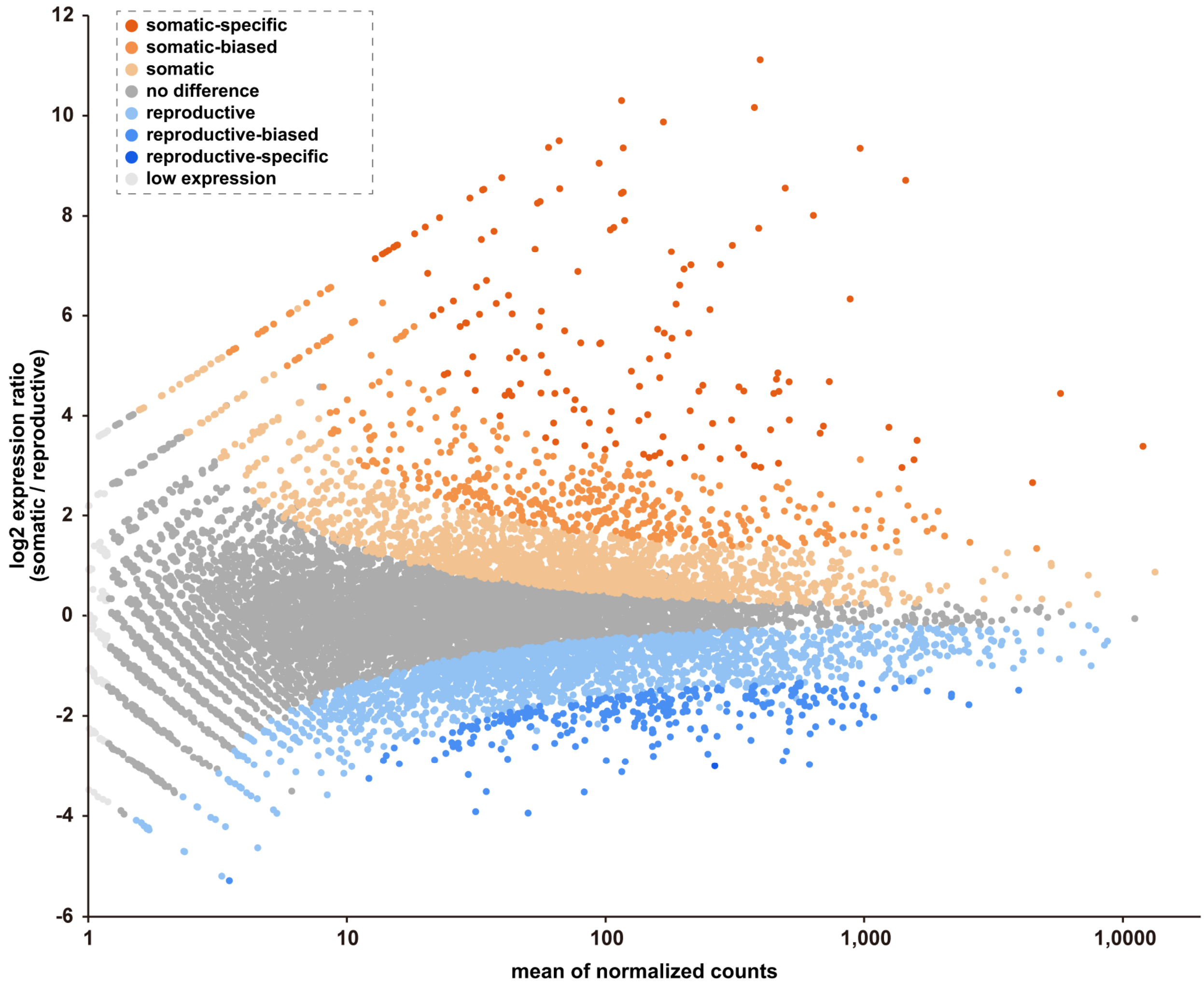

Supplementary Figure 9. MA plot of the cell-type RNA-seq analysis of *Astrephomene gubernaculifera*. Each dot represents a single gene with coloring by classification shown on upper-left. Mean of normalized read counts is plotted on x-axis, log scale. Ratio of somatic expression to reproductive expression calculated by DESeq2[60] is plotted on y-axis, log2 scale.

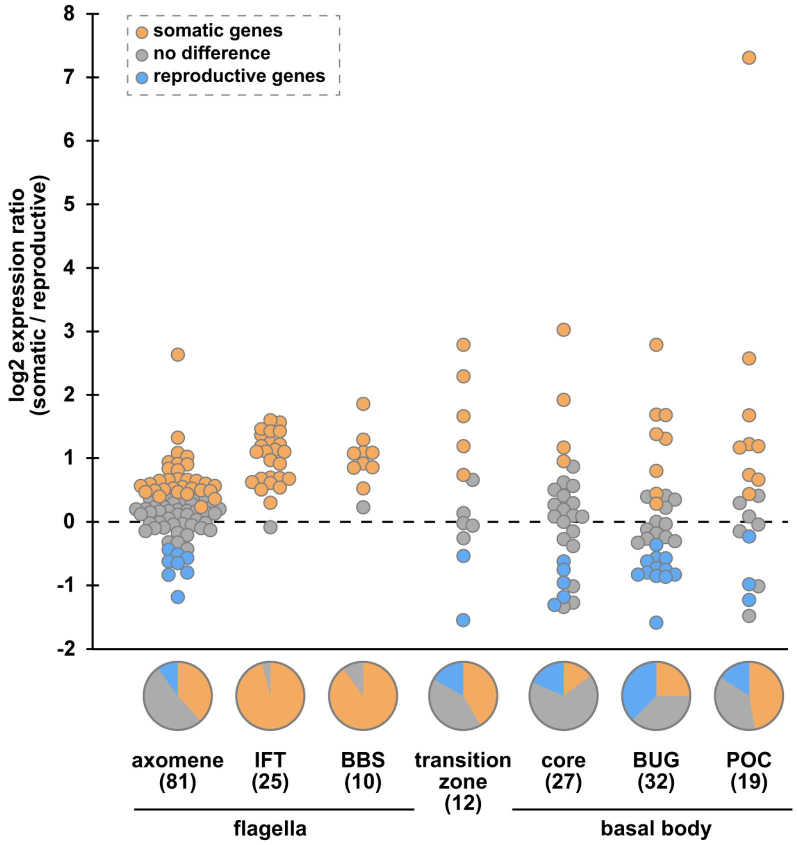

Supplementary Figure 10. Cell-type expression of flagella, transition zone and basal body genes in *Astrephomene gubernaculifera*. Expression ratio of axoneme genes, intraflagellar transport (IFT) genes, Bardet–Biedel Syndrome (BBS) genes, transition zone genes and basal bodies genes (core genes with validated function and localization, BUG genes and POC genes) (Supplementary Data 4) is shown. The number in parentheses indicates the number of genes in each category.

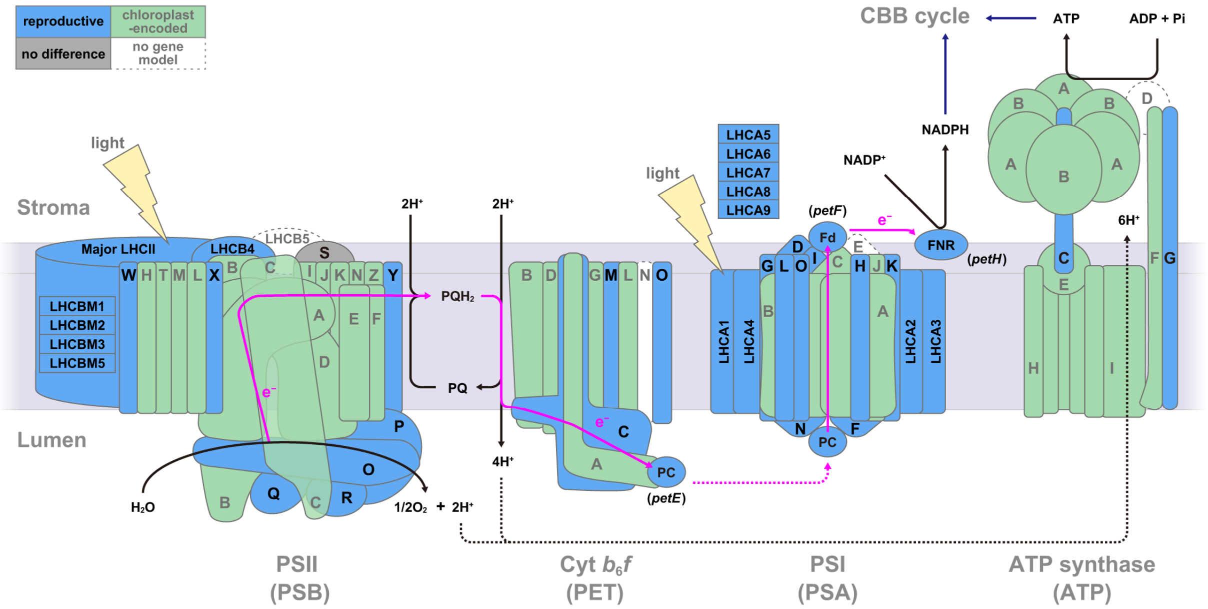

Supplementary Figure 11. Schematic diagram of complexes in photosynthetic light reactions and differentially expressed genes in *Astrephomene gubernaculifera*. See also Fig. 3c and Supplementary Data 5. The illustrated structure of the complexes is based on a previous study[68]. Each component is colored by classification shown on upper-left. Note the chloroplast-encoded gene in *A. gubernaculifera* (green) are also encoded in the chloroplast genome of *Chlamydomonas reinhardtii*[64].

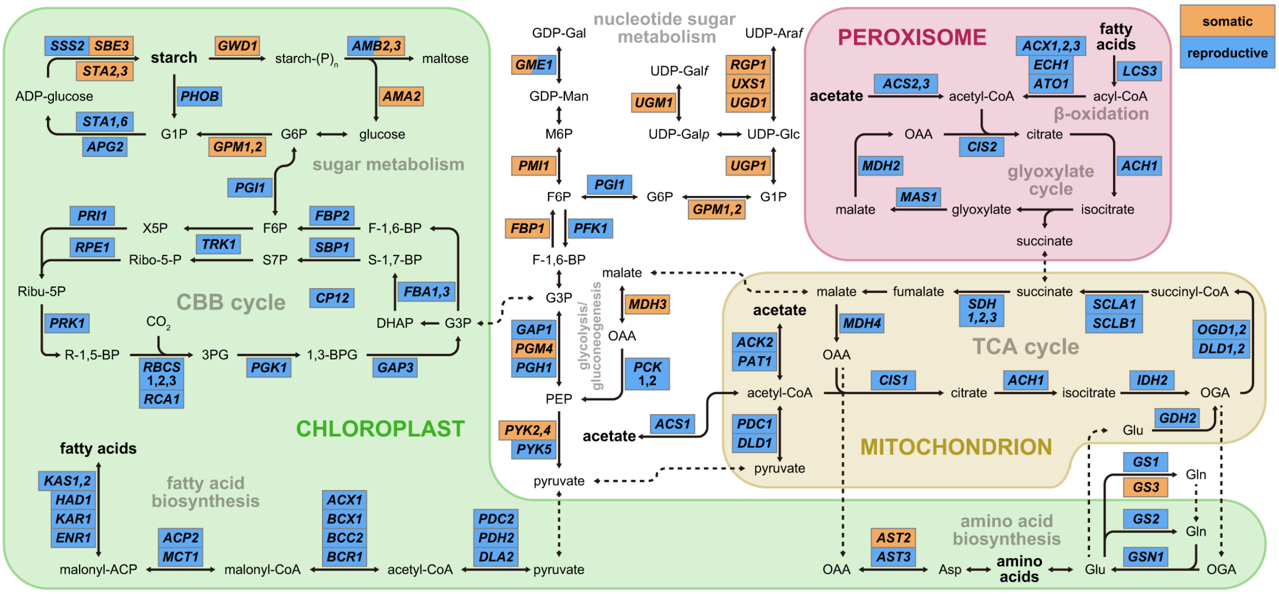

Supplementary Figure 12. Schematic diagram of carbon metabolic pathways and differentially expressed genes in *Astrephomene gubernaculifera*. See also Fig. 3d and Supplementary Data 6, S7. Solid arrows indicate chemical reactions, and dashed arrows indicate transportation of substrates between subcellular compartments. The metabolic pathways and the subcellular compartments (chloroplast, mitochondria, peroxisome and cytosol) are mainly based on previous studies[24,70,71] (Supplementary Methods). Each enzyme is colored by classification shown on upper-right.

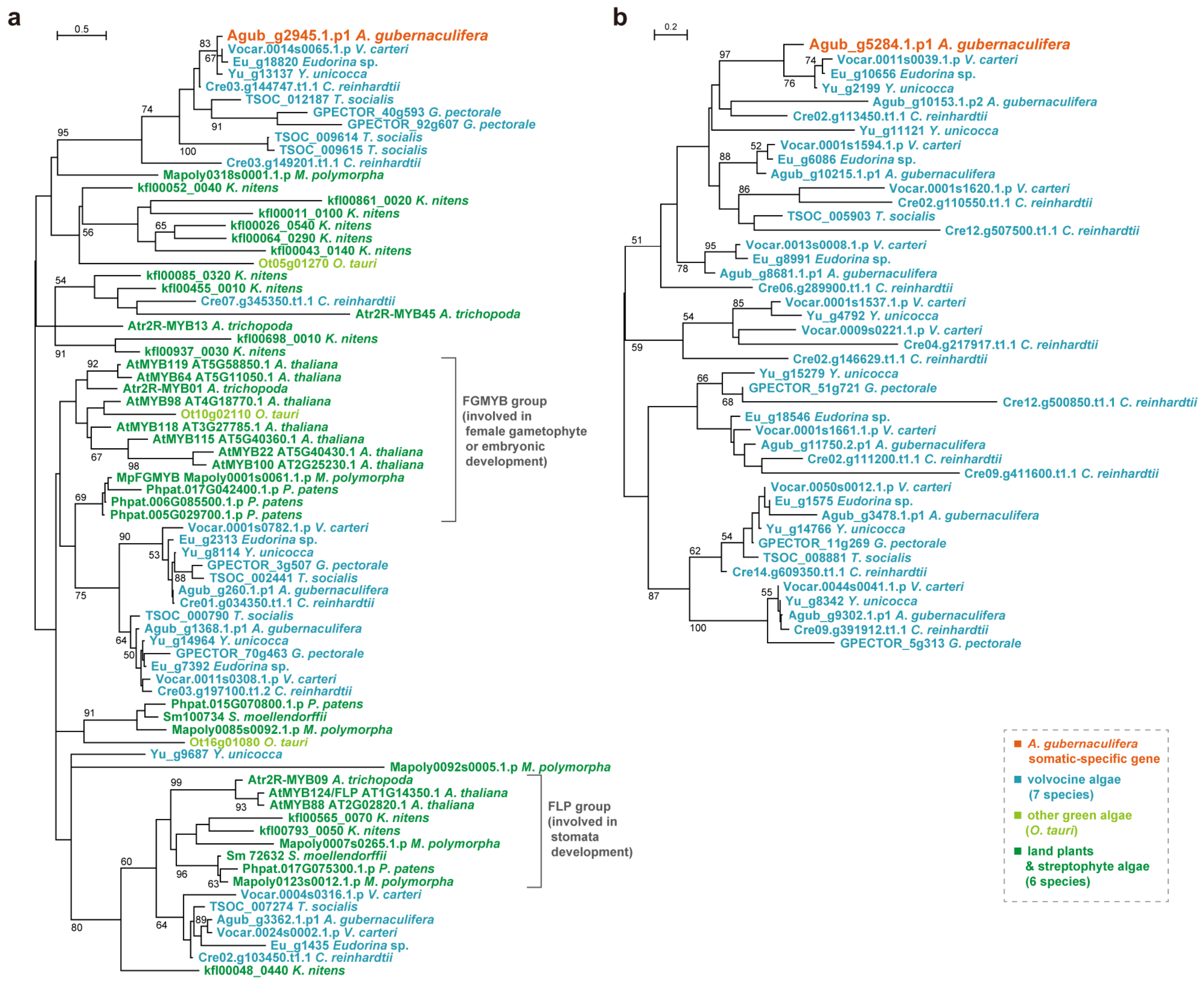

Supplementary Figure 13. Phylogenetic analysis of two somatic-specific MYB transcription factors in *Astrephomene gubernaculifera*. The original trees for Fig. 4b. (**a**) Phylogenetic tree of R2R3-MYB transcription factors including Agub_g2945. The 105 amino acid positions from DNA binding domain of 75 MYBs were subjected to maximum-likelihood analysis (LG + I + G model). The bootstrap values from maximum-likelihood (≥ 50%) are shown at branches. (**b**) Phylogenetic tree of 1R-MYB-like genes including Agub_g5284. The 87 amino acid positions from DNA binding domain of 43 MYBs were subjected to maximum-likelihood analysis (LG + I + G + F model). The bootstrap values from maximum-likelihood (≥ 50%) are shown at branches.

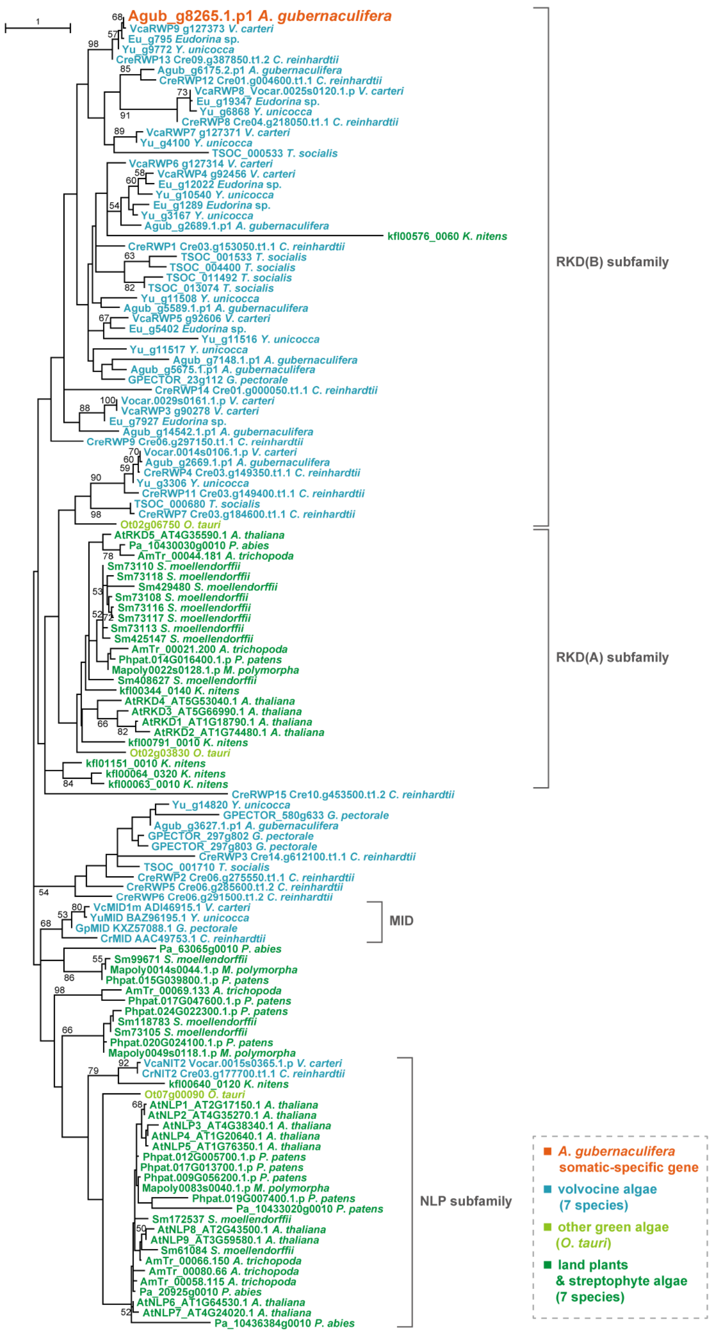

Supplementary Figure 14. Phylogenetic analysis of the somatic-specific RWP-RK transcription factor in *A. gubernaculifera*. The original tree for Fig. 4b. The 49 amino acid positions from 127 RWP-RK transcription factors including Agub_g8265 were subjected to maximum-likelihood analysis (LG + I + G model). The bootstrap values from maximum-likelihood (≥ 50%) are shown at branches.

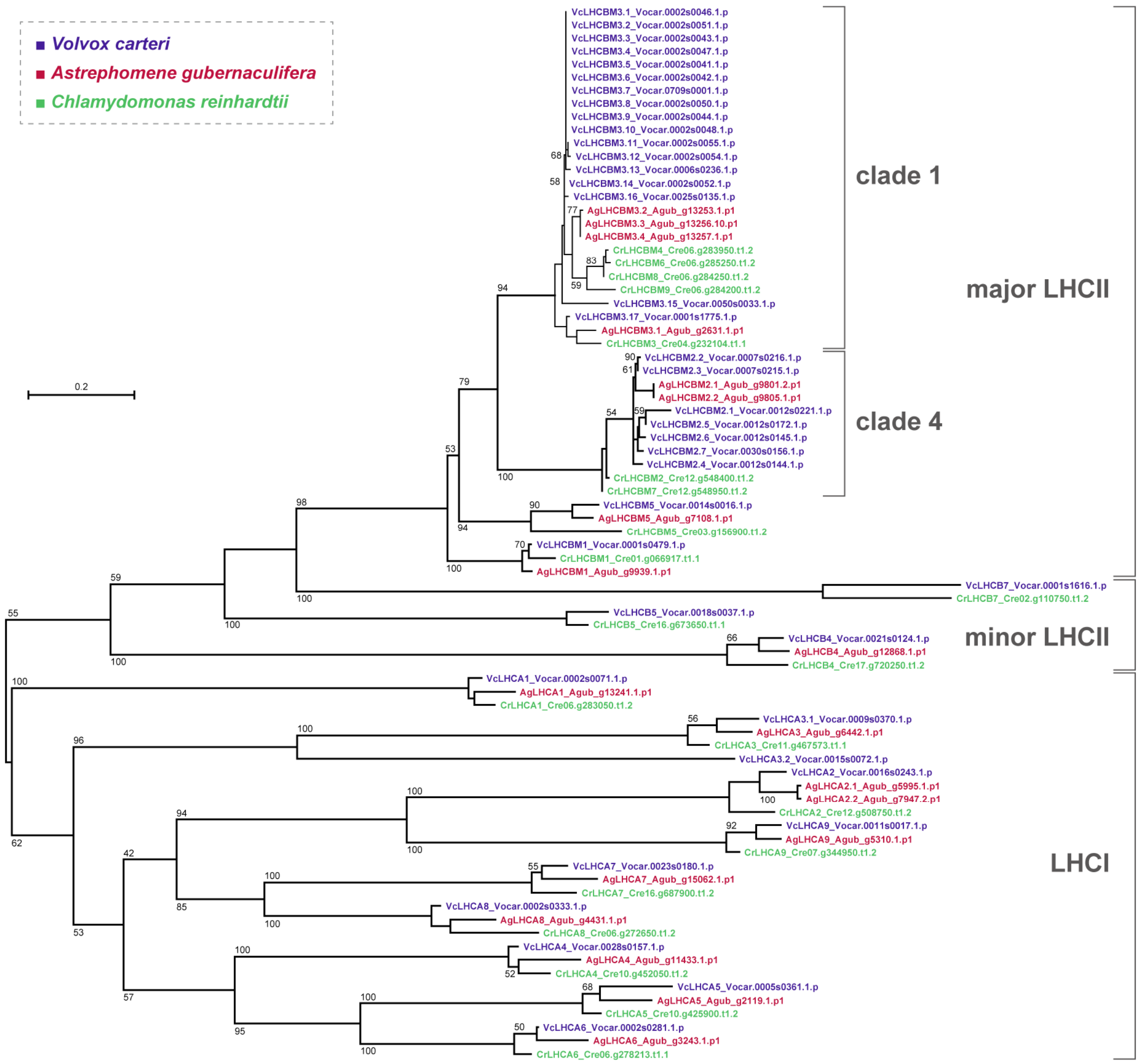

Supplementary Figure 15. Identification of LHC gene family in *Astrephomene gubernaculifera*. The 271 amino acid positions from LHCII and LHCI genes in *V. carteri*, *C. reinhardtii* and *A. gubernaculifera* (Supplementary Data 5) were subjected to maximum-likelihood analysis using LG + I + G + F model selected by ModelTest-NG[43] with 1,000 replicates of bootstrap analyses[44] by RAxML-NG[45]. The bootstrap values (≥ 50%) are shown at branches. The colors indicate species according to the color key at the upper left. The classification of LHCII genes are based on a previous study[24]. LHCB5 and LHCB7 are absent in gene models of *A. gubernaculifera* genome.

Supplementary References

1. Green, K. & Kirk, D. L. Cleavage patterns, cell lineages, and development of a cytoplasmic bridge system in *Volvox* embryos. *J. Cell Biol.* **91**, 743–755 (1981).

2. Viamontes, G. I. & Kirk, D. L. Cell shape changes and the mechanism of inversion in *Volvox*. *J. Cell Biol.* **75**, 719–730 (1977).

3. Kirk, D. L. & Nishii, I. *Volvox carteri* as a model for studying the genetic and cytological control of morphogenesis. *Dev. Growth Differ.* **43**, 621–631 (2001).

4. Nishii, I., Ogihara, S. & Kirk, D. L. A kinesin, InvA, plays an essential role in *Volvox* morphogenesis. *Cell* **113**, 743–753 (2003).

5. Ueki, N. & Nishii, I. *Idaten* is a new cold-inducible transposon of *Volvox carteri* that can be used for tagging developmentally important genes. *Genetics* **180**, 1343–1353 (2008).

6. Ueki, N. & Nishii, I. Controlled enlargement of the glycoprotein vesicle surrounding a *Volvox* embryo requires the InvB nucleotide-sugar transporter and is required for normal morphogenesis. *Plant Cell* **21**, 1166–1181 (2009).

7. Miller, S. M. & Kirk, D. L. *glsA*, a Volvox gene required for asymmetric division and germ cell specification, encodes a chaperone-like protein. *Development* **126**, 649–658 (1999).

8. Cheng, Q., Pappas, V., Hallmann, A. & Miller, S. M. Hsp70A and GlsA interact as partner chaperones to regulate asymmetric division in *Volvox*. *Dev. Biol.* **286**, 537–548 (2005).

9. Yamashita, S. *et al.* Alternative evolution of a spheroidal colony in volvocine algae: developmental analysis of embryogenesis in *Astrephomene* (Volvocales, Chlorophyta). *BMC Evol. Biol.* **16**, 243 (2016).

10. Hallmann, A. Extracellular matrix and sex-inducing pheromone in *Volvox*. *Int. Rev. Cytol.* **227**, 131–182 (2003).

11. Prochnik, S. E. *et al.* Genomic analysis of organismal complexity in the multicellular green alga *Volvox carteri*. *Science* **329**, 223–226 (2010).

12. Hanschen, E. R. *et al.* The *Gonium pectorale* genome demonstrates co-option of cell cycle regulation during the evolution of multicellularity. *Nat. Commun.* **7**, 11370 (2016).

13. Featherston, J. *et al.* The 4-celled *Tetrabaena socialis* nuclear genome reveals the essential components for genetic control of cell number at the origin of multicellularity in the volvocine lineage. *Mol. Biol. Evol.* **35**, 855–870 (2018).

14. Hoops, H. J. & Floyd, G. L. Mitosis, cytokinesis and colony formation in the colonial green alga *Astrephomene gubernaculifera*. *Br. Phycol. J.* **17**, 297–310 (1982).

15. Nozaki, H. Ultrastructure of the extracellular matrix of *Gonium* (Volvocales, Chlorophyta). *Phycologia* **29**, 1–8 (1990).

16. Herron, M. D. & Michod, R. E. Evolution of complexity in the volvocine algae: transitions in individuality through Darwin's eye. *Evolution* **62**, 436–451 (2008).

17. Herron, M. D., Hackett, J. D., Aylward, F. O. & Michod, R. E. Triassic origin and early radiation of multicellular volvocine algae. *Proc. Natl. Acad. Sci. U. S. A.* **106**, 3254–3258 (2009).

18. Lindsey, C. R., Rosenzweig, F. & Herron, M. D. Phylotranscriptomics points to multiple independent origins of multicellularity and cellular differentiation in the volvocine algae. *BMC Biol.* **19**, 182 (2021).

19. Nozaki, H. & Kuroiwa, T. Ultrastructure of the extracellular matrix and taxonomy of *Eudorina*, *Pleodorina* and *Yamagishiella* gen. nov. (Volvocaceae, Chlorophyta). *Phycologia* **31**, 529–541 (1992).

20. Sumper, M., Berg, E., Wenzl, S. & Godl, K. How a sex pheromone might act at a concentration below 10(−16) M. *EMBO J.* **12**, 831–836 (1993).

21. Godl, K., Hallmann, A., Rappel, A. & Sumper, M. Pherophorins: a family of extracellular matrix glycoproteins from *Volvox* structurally related to the sex-inducing pheromone. *Planta* **196**, 781–787 (1995).

22. Kirk, D. L. A twelve-step program for evolving multicellularity and a division of labor. *BioEssays* **27**, 299–310 (2005).

23. Hamaji, T. *et al.* Anisogamy evolved with a reduced sex-determining region in volvocine green algae. *Commun. Biol.* **1**, 17 (2018).

24. Matt, G. Y. & Umen, J. G. Cell-type transcriptomes of the multicellular green alga *Volvox carteri* yield insights into the evolutionary origins of germ and somatic differentiation programs. *Genes|Genomes|Genetics* **8**, 531–550 (2018).

25. Nematollahi, G., Kianianmomeni, A. & Hallmann, A. Quantitative analysis of cell-type specific gene expression in the green alga *Volvox carteri*. *BMC Genomics* **7**, 321 (2006).

26. Kawachi, M. *et al.* MCC-NIES list of strains, 9th edition, microbial culture collection at National Institute for Environmental Studies, Tsukuba, Japan. https://mcc.nies.go.jp/download/list9th_e.pdf (2013).

27. Kajitani, R. *et al.* Efficient de novo assembly of highly heterozygous genomes from whole-genome shotgun short reads. *Genome Res.* **24**, 1384–1395 (2014).

28. Gaouda, H. *et al.* Exploring the limits and causes of plastid genome expansion in volvocine green algae. *Genome Biol. Evol.* **10**, 2248–2254 (2018).

29. Altschul, S. F., Gish, W., Miller, W., Myers, E. W. & Lipman, D. J. Basic local alignment search tool. *J. Mol. Biol.* **215**, 403–410 (1990).

30. Chaisson, M. J. & Tesler, G. Mapping single molecule sequencing reads using basic local alignment with successive refinement (BLASR): application and theory. *BMC Bioinformatics* **13**, 238 (2012).

31. Koren, S. *et al.* Canu: scalable and accurate long-read assembly via adaptive *k* -mer weighting and repeat separation. *Genome Res.* **27**, 722–736 (2017).

32. Zimin, A. V. *et al.* The MaSuRCA genome assembler. *Bioinformatics* **29**, 2669–2677 (2013).

33. Walker, B. J. *et al.* Pilon: an integrated tool for comprehensive microbial variant detection and

genome assembly improvement. *PLOS ONE* **9**, e112963 (2014).

34. Tillich, M. *et al.* GeSeq – versatile and accurate annotation of organelle genomes. *Nucleic Acids Res.* **45**, W6–W11 (2017).

35. Emms, D. M. & Kelly, S. OrthoFinder: solving fundamental biases in whole genome comparisons dramatically improves orthogroup inference accuracy. *Genome Biol.* **16**, 157 (2015).

36. Edgar, R. C. MUSCLE: multiple sequence alignment with high accuracy and high throughput. *Nucleic Acids Res.* **32**, 1792–1797 (2004).

37. Suyama, M., Torrents, D. & Bork, P. PAL2NAL: robust conversion of protein sequence alignments into the corresponding codon alignments. *Nucleic Acids Res.* **34**, W609–W612 (2006).

38. Yang, Z. PAML 4: Phylogenetic Analysis by Maximum Likelihood. *Mol. Biol. Evol.* **24**, 1586–1591 (2007).

39. Flynn, J. M. *et al.* RepeatModeler2 for automated genomic discovery of transposable element families. *Proc. Natl. Acad. Sci.* **117**, 9451–9457 (2020).

40. Grabherr, M. G. *et al.* Full-length transcriptome assembly from RNA-Seq data without a reference genome. *Nat. Biotechnol.* **29**, 644–652 (2011).

41. Nakamura, T., Yamada, K. D., Tomii, K. & Katoh, K. Parallelization of MAFFT for large-scale multiple sequence alignments. *Bioinformatics* **34**, 2490–2492 (2018).

42. Capella-Gutierrez, S., Silla-Martinez, J. M. & Gabaldon, T. trimAl: a tool for automated alignment trimming in large-scale phylogenetic analyses. *Bioinformatics* **25**, 1972–1973 (2009).

43. Darriba, D. *et al.* ModelTest-NG: a new and scalable tool for the selection of DNA and protein evolutionary models. *Mol. Biol. Evol.* **37**, 291–294 (2020).

44. Felsenstein, J. Confidence limits on phylogenies: an approach using the bootstrap. *Evolution* **39**, 783–791 (1985).

45. Kozlov, A. M., Darriba, D., Flouri, T., Morel, B. & Stamatakis, A. RAxML-NG: a fast, scalable and user-friendly tool for maximum likelihood phylogenetic inference. *Bioinformatics* **35**, 4453–4455 (2019).

46. Ronquist, F. *et al.* MrBayes 3.2: Efficient Bayesian phylogenetic inference and model choice across a large model space. *Syst. Biol.* **61**, 539–542 (2012).

47. Kirk, M. M. *et al. regA*, a *Volvox* gene that plays a central role in germ-soma differentiation, encodes a novel regulatory protein. *Development* **126**, 639–647 (1999).

48. Duncan, L. *et al.* The *VARL* gene family and the evolutionary origins of the master cell-type regulatory gene, *regA*, in *Volvox carteri*. *J. Mol. Evol.* **65**, 1–11 (2007).

49. Grochau-Wright, Z. I. *et al.* Genetic basis for soma is present in undifferentiated volvocine green algae. *J. Evol. Biol.* **30**, 1205–1218 (2017).

50. Kumar, S., Stecher, G. & Tamura, K. MEGA7: Molecular Evolutionary Genetics Analysis version 7.0 for bigger datasets. *Mol. Biol. Evol.* **33**, 1870–1874 (2016).

51. Hanschen, E. R., Ferris, P. J. & Michod, R. E. Early evolution of the genetic basis for soma in the Volvocaceae: evolution of the genetic basis for soma. *Evolution* **68**, 2014–2025 (2014).

52. Cheng, Q., Hallmann, A., Edwards, L. & Miller, S. M. Characterization of a heat-shock-inducible *hsp70* gene of the green alga *Volvox carteri*. *Gene* **371**, 112–120 (2006).

53. Nozaki, H., Kuroiwa, H., Mita, T. & Kuroiwa, T. *Pleodorina japonica* sp. nov. (Volvocales, Chlorophyta) with bacteria-like endosymbionts. *Phycologia* **28**, 252–267 (1989).

54. Nozaki, H. *et al.* Origin and evolution of the colonial Volvocales (Chlorophyceae) as inferred from multiple, chloroplast gene sequences. *Mol. Phylogenet. Evol.* **17**, 256–268 (2000).

55. Godl, K., Hallmann, A., Wenzl, S. & Sumper, M. Differential targeting of closely related ECM glycoproteins: the pherophorin family from *Volvox*. *EMBO J.* **16**, 25–34 (1997).

56. Bolger, A. M., Lohse, M. & Usadel, B. Trimmomatic: a flexible trimmer for Illumina sequence data. *Bioinformatics* **30**, 2114–2120 (2014).

57. Schmieder, R. & Edwards, R. Quality control and preprocessing of metagenomic datasets. *Bioinformatics* **27**, 863–864 (2011).

58. Kim, D., Paggi, J. M., Park, C., Bennett, C. & Salzberg, S. L. Graph-based genome alignment and genotyping with HISAT2 and HISAT-genotype. *Nat. Biotechnol.* **37**, 907–915 (2019).

59. Liao, Y., Smyth, G. K. & Shi, W. featureCounts: an efficient general purpose program for assigning sequence reads to genomic features. *Bioinformatics* **30**, 923–930 (2014).

60. Love, M. I., Huber, W. & Anders, S. Moderated estimation of fold change and dispersion for RNA-seq data with DESeq2. *Genome Biol.* **15**, 550 (2014).

61. Klein, B., Wibberg, D. & Hallmann, A. Whole transcriptome RNA-Seq analysis reveals extensive cell type-specific compartmentalization in *Volvox carteri*. *BMC Biol.* **15**, (2017).

62. Wagner, G. P., Kin, K. & Lynch, V. J. Measurement of mRNA abundance using RNA-seq data: RPKM measure is inconsistent among samples. *Theory Biosci.* **131**, 281–285 (2012).

63. Conesa, A. *et al.* Blast2GO: a universal tool for annotation, visualization and analysis in functional genomics research. *Bioinformatics* **21**, 3674–3676 (2005).

64. Buchfink, B., Xie, C. & Huson, D. H. Fast and sensitive protein alignment using DIAMOND. *Nat. Methods* **12**, 59–60 (2015).

65. Quevillon, E. *et al.* InterProScan: protein domains identifier. *Nucleic Acids Res.* **33**, W116–W120 (2005).

66. Myhre, S., Tveit, H., Mollestad, T. & Laegreid, A. Additional Gene Ontology structure for improved biological reasoning. *Bioinformatics* **22**, 2020–2027 (2006).

67. Camon, E. The Gene Ontology Annotation (GOA) Database: sharing knowledge in Uniprot with Gene Ontology. *Nucleic Acids Res.* **32**, 262D – 266 (2004).

68. Allen, J. F., de Paula, W. B. M., Puthiyaveetil, S. & Nield, J. A structural phylogenetic map for chloroplast photosynthesis. *Trends Plant Sci.* **16**, 645–655 (2011).

69. Barker, A. R., Renzaglia, K. S., Fry, K. & Dawe, H. R. Bioinformatic analysis of ciliary

transition zone proteins reveals insights into the evolution of ciliopathy networks. *BMC Genomics* **15**, 531 (2014).

70. Zones, J. M., Blaby, I. K., Merchant, S. S. & Umen, J. G. High-resolution profiling of a synchronized diurnal transcriptome from *Chlamydomonas reinhardtii* reveals continuous cell and metabolic differentiation. *Plant Cell* **27**, 2743–2769 (2015).

71. Johnson, X. & Alric, J. Central carbon metabolism and electron transport in *Chlamydomonas reinhardtii*: metabolic constraints for carbon partitioning between oil and starch. *Eukaryot. Cell* **12**, 776–793 (2013).

72. Chardin, C., Girin, T., Roudier, F., Meyer, C. & Krapp, A. The plant RWP-RK transcription factors: key regulators of nitrogen responses and of gametophyte development. *J. Exp. Bot.* **65**, 5577–5587 (2014).

73. Du, H. *et al.* The evolutionary history of R2R3-MYB proteins across 50 eukaryotes: new insights into subfamily classification and expansion. *Sci. Rep.* **5**, 11037 (2015).

74. Bowman, J. L. *et al.* Insights into land plant evolution garnered from the *Marchantia polymorpha* genome. *Cell* **171**, 287-304.e15 (2017).

75. Merchant, S. S. *et al.* The *Chlamydomonas* genome reveals the evolution of key animal and plant functions. *Science* **318**, 245–250 (2007).

76. Stanke, M., Diekhans, M., Baertsch, R. & Haussler, D. Using native and syntenically mapped cDNA alignments to improve *de novo* gene finding. *Bioinformatics* **24**, 637–644 (2008).

77. Yamamoto, K. *et al*. Three genomes in the algal genus *Volvox* reveal the fate of a haploid sex-determining region after a transition to homothallism. *Proc. Natl. Acad. Sci. U. S. A.* **118**, e2100712118 (2021).

78. Craig, R. J., Hasan, A. R., Ness, R. W. & Keightley, P. D. Comparative genomics of *Chlamydomonas*. *Plant Cell* **33**, 1016–1041 (2021).

79. Lamesch, P. *et al.* The Arabidopsis Information Resource (TAIR): improved gene annotation

and new tools. *Nucleic Acids Res.* **40**, D1202–D1210 (2012).

80. Albert, V. A. *et al.* The *Amborella* genome and the evolution of flowering plants. *Science* **342**, 1241089 (2013).

81. Nystedt, B. *et al.* The Norway spruce genome sequence and conifer genome evolution. *Nature* **497**, 579–584 (2013).

82. Banks, J. A. *et al.* The Selaginella genome identifies genetic changes associated with the evolution of vascular plants. *Science* **332**, 960–963 (2011).

83. Rensing, S. A. *et al.* The *Physcomitrella* genome reveals evolutionary insights into the conquest of land by plants. *Science* **319**, 64–69 (2008).

84. Hori, K. *et al. Klebsormidium flaccidum* genome reveals primary factors for plant terrestrial adaptation. *Nat. Commun.* **5**, 3978 (2014).

85. Derelle, E. *et al.* Genome analysis of the smallest free-living eukaryote *Ostreococcus tauri* unveils many unique features. *Proc. Natl. Acad. Sci. U. S. A.* **103**, 11647–11652 (2006).

86. Matsuzaki, M. *et al.* Genome sequence of the ultrasmall unicellular red alga *Cyanidioschyzon merolae* 10D. *Nature* **428**, 653–657 (2004).

87. Greiner, S., Lehwark, P. & Bock, R. OrganellarGenomeDRAW (OGDRAW) version 1.3.1: expanded toolkit for the graphical visualization of organellar genomes. *Nucleic Acids Res.* **47**, W59–W64 (2019).
